# Breaking the ORR Trade‐Off via Mg‐Steered Fe‐N_4_ Pyridinic Conversion

**DOI:** 10.1002/smll.202514722

**Published:** 2026-02-04

**Authors:** Si‐Qi Sun, Ya‐Peng Cheng, Hai‐Ning Zhang, Jia‐Min Lyu, Xiao‐Yun Li, Ke Lyu, Ming‐Hui Sun, Shen Yu, Wen‐Mao Tu, Andreu Cabot, Li‐Hua Chen

**Affiliations:** ^1^ State Key Laboratory of Advanced Technology for Materials Synthesis and Processing Wuhan University of Technology Wuhan P. R. China; ^2^ Catalonia Institute for Energy Research‐IREC Jardins de les Dones de Negre Sant Adrià de Besòs Catalonia Spain; ^3^ Universitat de Barcelona Martí i Franquès 1 Barcelona Catalonia Spain; ^4^ Hubei Key Laboratory of Fuel Cell Wuhan University of Technology Wuhan P. R. China; ^5^ State Key Laboratory of Silicate Materials for Architectures Wuhan University of Technology Wuhan P. R. China; ^6^ ICREA Pg. Lluis Company Barcelona Catalonia Spain

**Keywords:** coordination environment, Fe‐N_4_, oxygen reduction reaction, single atom catalyst, Zn‐air Battery

## Abstract

Single‐atom Fe sites coordinated by pyrrolic nitrogen (Fe‐N_pyrr_‐C) are highly active for the oxygen reduction reaction (ORR) but suffer from rapid demetalization‐induced deactivation. Here, we overcome this limitation via a Mg‐assisted sacrificial templating strategy that precisely reconstructs Fe‐N_4_ coordination, driving partial conversion of unstable pyrrolic‐N to robust pyridinic‐N ligands. The resulting Fe(Mg)‐N‐C(1) catalyst exhibits exceptional ORR performance (half‐wave potential E_1/2_ = 0.91 V) and outstanding durability, retaining 95.2% of its initial current after 55 h, surpassing both Fe‐N‐C and Pt/C. In Zn‐air batteries, it enables stable operation for > 530 h (peak power density: 271 mW cm^−^^2^). This work demonstrates that enriching pyridinic‐N‐coordinated Fe‐N_4_ sites simultaneously enhances activity and suppresses demetalization, offering a general coordination‐engineering strategy to unify activity and stability in Fe‐N‐C electrocatalysts.

## Graphical abstract

A Mg‐assisted sacrificial templating strategy is developed to selectively regulate the coordination environment of Fe–N_4_ sites. This approach induces an active site conversion from S1 to S2, enabling enhancement of oxygen reduction activity and durability.

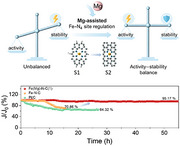

## 1 Introduction

Proton‐exchange membrane fuel cells (PEMFCs) and electrolyzers remain heavily dependent on Pt‐based catalysts [1, 2, 3, 4], whose prohibitive cost constitutes a major barrier to large‐scale PEMFC deployment [5, 6, 7, 8]. Consequently, intensive research has focused on earth‐abundant alternatives. Among non‐precious‐metal systems, metal‐nitrogen‐carbon (M‐N‐C) materials [9, 10, 11, 12] particularly iron‐nitrogen‐carbon (Fe‐N‐C)—are widely regarded as the most promising Pt replacements [13, 14, 15]. These catalysts exhibit exceptional activity for critical energy‐conversion reactions including the oxygen reduction reaction (ORR), carbon dioxide reduction reaction (CO_2_RR), and nitrogen reduction reaction (NRR) [15, 16, 17, 18, 19, 20].

The remarkable activity of Fe‐N‐C catalysts is primarily attributed to atomically dispersed Fe‐N_4_ sites [21], whose intrinsic properties are governed by the electronic and structural environment imposed by their four nitrogen ligands within the graphitic carbon matrix [22]. Extensive efforts have therefore explored levers to optimize activity, such as tuning surface area, porosity, graphitization degree, Fe‐N_x_ coordination geometry, and carbon scaffold electronic structure [23, 24, 25, 26, 27, 28, 29, 30, 31, 32, 33]. A pivotal advancement has been elucidating how distinct nitrogen coordination motifs dictate ORR performance. Jaouen et al. identified two dominant FeN_4_ architectures: FeN_4_C_12_ (with pyrrolic‐N coordination) and FeN_4_C_10_ (with pyridinic‐N ligands) [34]. Compared to FeN_4_C_10_, the more open local carbon environment in FeN_4_C_12_ strengthens electronic localization and enhances adsorption of oxygenated intermediates, thereby optimizing the ORR pathway [34]. This structure‐activity framework provides a rational foundation for engineering high‐performance Fe‐N_4_ sites.

Despite its superior activity, the FeN_4_C_12_ moiety suffers from irreversible degradation during ORR, driven primarily by demetalization of Fe centers and carbon oxidation reactions (COR) [23, 24, 25, 26]. Catalyst deactivation stems from weakened Fe─N bonding at edge‐rich pyrrolic sites, where competition with protons accelerates Fe leaching [35], while COR destabilizes the carbon support matrix, triggering Fe loss and structural collapse [23, 24, 25, 26]. Although strategies like converting pyrrolic to pyridinic configurations [36] or enhancing graphitization [37] improve stability, simultaneously achieving high activity and long‐term operational durability remains an unresolved challenge.

To bridge this critical gap, we introduce a Mg‐driven sacrificial templating strategy that directly reconstructs Fe‐N_4_ coordination chemistry. During high‐temperature etching with Mg/NH_4_Cl, neighboring N and C atoms become dynamically mobile, enabling the partial conversion of unstable pyrrolic Fe‐N_4_ sites (S1) into robust pyridinic Fe‐N_4_ configurations (S2). This targeted reconfiguration suppresses Fe demetalization and reinforces structural integrity while preserving catalytic activity. The optimized catalyst delivers exceptional ORR stability alongside high performance, enabling Zn‐air batteries with > 530 h operational longevity and 271 mW cm^−^^2^ peak power density. Our work establishes coordination engineering through sacrificial templating as a universal pathway to unify activity and stability in M‐N‐C electrocatalysts.

## 2 Results and Discussion

### 2.1 Electrocatalyst Synthesis and Structural Characterization

**FIGURE 1:**
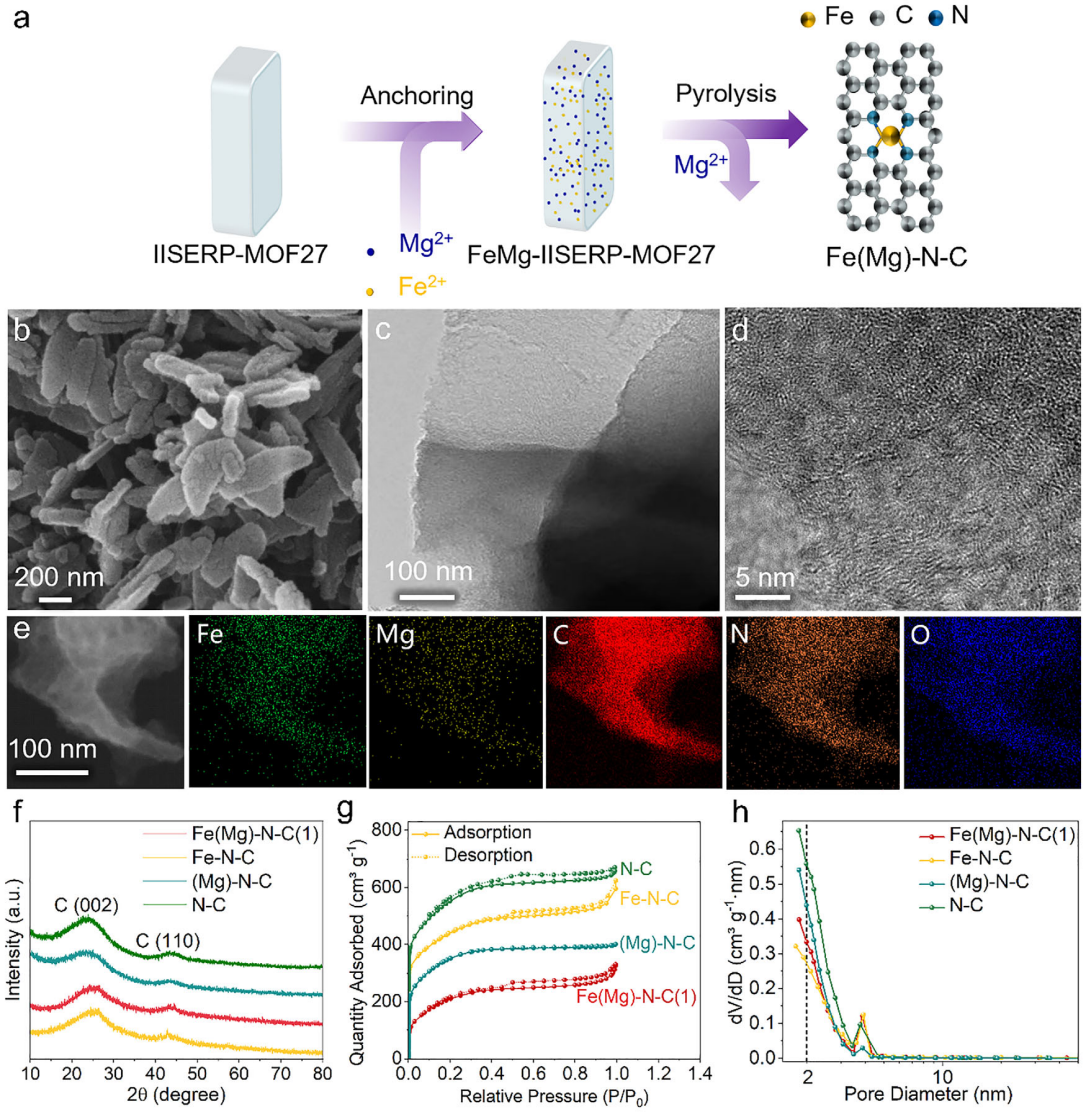
Synthesis method and morphological characterization. (a) Schematic illustration of the synthesis of the Fe(Mg)‐N‐C(1) catalyst. (b) SEM image, (c) TEM image, (d) HAADF‐STEM image, and (e) elemental mapping of Fe(Mg)‐N‐C(1). (f) XRD patterns of Fe(Mg)‐N‐C(1), Fe‐N‐C, (Mg)‐N‐C and N‐C. (g) N_2_ adsorption/desorption and (h) pore distribution plots of Fe(Mg)‐N‐C(1), Fe‐N‐C, (Mg)‐N‐C and N‐C.

The fabrication process is depicted in Figure 1a. A crystalline IISERP‐MOF27 precursor was synthesized via solvothermal reaction of zinc nitrate, adenine, and terephthalic acid [38, 39]. This metal‐organic framework (MOF) provides a dense N/O‐donor environment that anchors metal ions and suppresses aggregation at elevated temperatures. Specifically, accessible ─NH_2_ groups on adenine and ─COOH moieties of terephthalate coordinate with Fe^3^^+^ and Mg^2^^+^ introduced during a subsequent hydrothermal step, yielding the FeMg‐IISERP‐MOF27(*x*) composite, the variable *x* denotes the molar ratio of n(Mg) to n(Fe) in the metal precursor [39]. Subsequent NH_4_Cl‐assisted pyrolysis (900°C) incorporates atomically dispersed Fe into the carbon framework, while Mg is predominantly converted to MgO [38, 39]. During pyrolysis, NH_4_Cl decomposes to NH_3_ and HCl. The in situ‐generated HCl etches away MgO, while concurrent activation of N/C species drives partial reconstruction of pyrrolic‐N‐coordinated Fe‐N_4_ sites (S1) into more stable pyridinic‐N‐coordinated Fe‐N_4_ sites (S2) [36]. This Mg‐induced reconstruction thereby biases the active‐site distribution toward the pyridinic configuration. For clarity, the resultant materials are denoted as Fe(Mg)‐N‐C(*x*) (derived from FeMg‐IISERP‐MOF27(*x*)), Fe‐N‐C (from Fe‐IISERP‐MOF27), (Mg)‐N‐C (from Mg‐IISERP‐MOF27), and N‐C (from IISERP‐MOF27).

As evidenced by Figure 1b and Figure S2, Fe(Mg)‐N‐C(1) maintains the flake‐like morphology of its MOF precursor. High‐resolution transmission electron microscopy (TEM) confirms retention of a partially graphitized carbon framework in Fe‐N‐C, with morphology closely mirroring that of the parent MOF (Figure 1c, Figure S3). This structural consistency is further corroborated by X‐ray diffraction (XRD) analysis (Figure 1f), where two broad reflections at ≈26.2° and 43.7° correspond to the (002) and (100) planes of graphitic carbon. Critically, the absence of diffraction peaks attributable to metallic Fe phases confirms atomic dispersion of Fe species rather than aggregation into crystalline nanoparticles.

Inductively coupled plasma optical emission spectrometry (ICP‐OES) analysis confirmed Fe and Mg loadings of 3.94 wt.% and 0.03 wt.%, respectively, in Fe(Mg)‐N‐C(1) (Table S1). To elucidate NH_4_Cl's role, a control synthesis was conducted without NH_4_Cl addition during carbonization. The resultant catalyst exhibited significantly higher Mg residue (1.93 wt.%) and reduced Fe content (2.85 wt.%) (Table S2), demonstrating that gaseous decomposition products (HCl/NH_3_) from NH_4_Cl effectively remove Mg species. The moderately elevated Fe loading in the NH_4_Cl‐assisted sample arises from supplementary nitrogen that reinforces Fe anchoring within the carbon matrix.

Nitrogen adsorption‐desorption analyses further probed the catalysts' textural properties. The Fe(Mg)‐N‐C(1) catalyst exhibits a moderately lower specific surface area (998 m^2^ g^−^^1^) than Fe‐N‐C (1146 m^2^ g^−^^1^), as shown in Figure 1g,h and Table S3. It was also observed that the proportion of micropores in Fe(Mg)‐N‐C(1) decreased (Table S4). This reduction likely stems from the Fe‐N_4_ site reconstruction, wherein conversion from the open pyrrolic configuration to the compact in‐plane pyridinic geometry marginally diminishes accessible surface area. Crucially, Fe(Mg)‐N‐C(1) maintains substantial hierarchical porosity spanning multiple length scales—a feature highly favorable for efficient mass transport during electrochemical processes.

**FIGURE 2:**
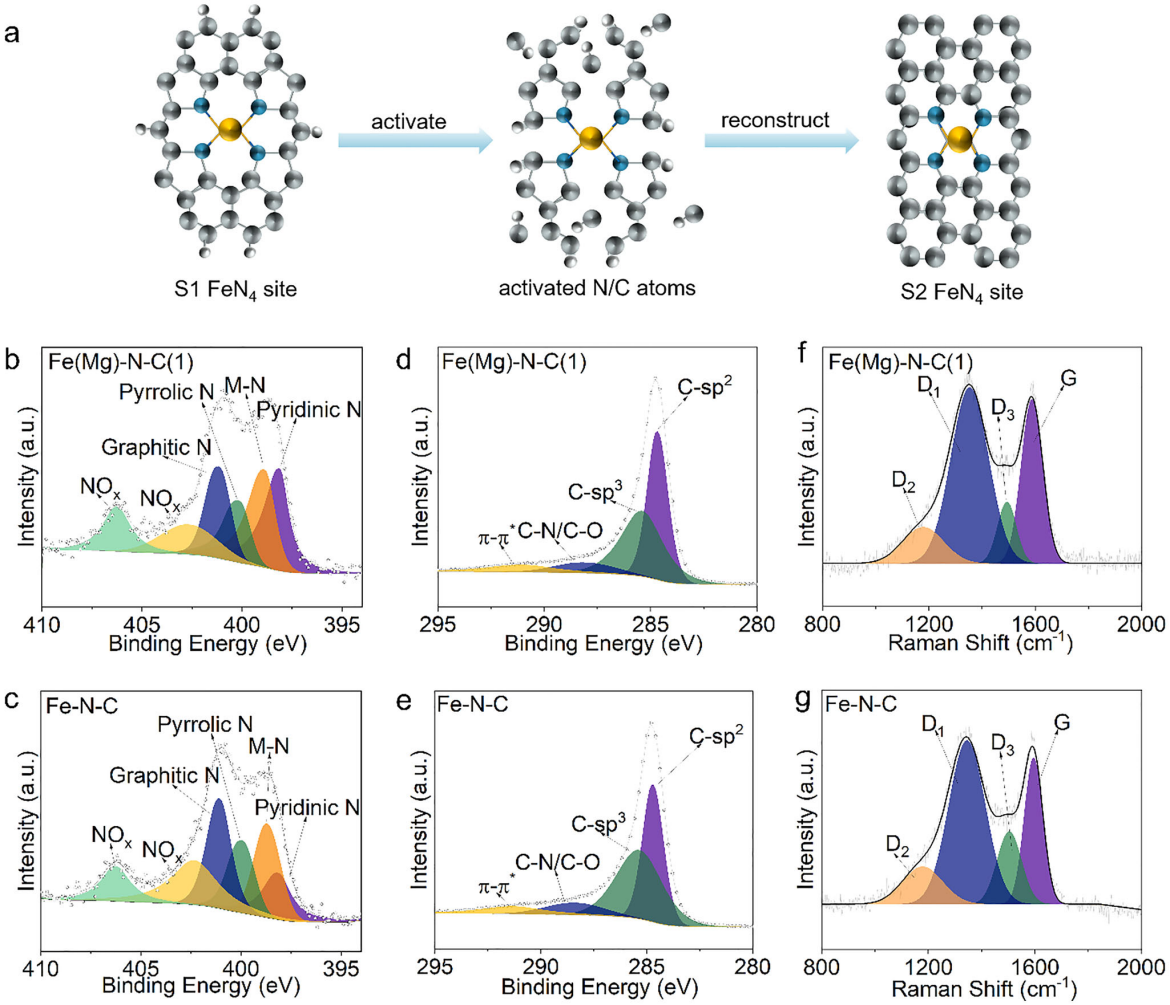
Structural characterization. (a) Possible structural evolution pathway from an S1 to an S2 site. The grey, blue, white, and orange balls represent C, N, H, and Fe atoms, respectively. (b,c) High‐resolution N 1s XPS spectra of Fe(Mg)‐N‐C(1) and Fe‐N‐C. (d,e) High‐resolution C 1s XPS spectra of Fe(Mg)‐N‐C(1) and Fe‐N‐C. (f,g) Raman spectra for Fe(Mg)‐N‐C(1) and Fe‐N‐C.

The conversion pathway from pyrrolic Fe‐N_4_ sites (S1) to pyridinic Fe‐N_4_ sites (S2) is schematically depicted in Figure 2a. Under nitrogen‐rich pyrolysis conditions, Fe exhibits preferential retention within the carbon matrix. Critically, introduced Mg facilitates N/C vacancy formation and activates adjacent N/C atoms. This dynamic activation enables thermal reconstruction of active sites toward more stable graphitic configurations, corresponding to the S1 → S2 transition. X‐ray photoelectron spectroscopy (XPS) analysis reveals compositional evolution across catalysts. High‐resolution N 1s spectra of Fe(Mg)‐N‐C(1) and Fe‐N‐C (Figure 2b,c) resolve six distinct components: pyridinic‐N (398.2 eV), pyrrolic‐N (399.8 eV), graphitic‐N (401.1 eV), metal‐N_x_ (398.7 eV), and oxidized‐N (>402 eV) [40]. Fe(Mg)‐N‐C(1) demonstrates significantly enriched pyridinic‐N content, while Fe‐N‐C shows predominant pyrrolic‐N—directly evidencing S1 → S2 redistribution. The definitive metal‐N_x_ signature confirms Fe‐N coordination integrity. This nitrogen‐modulation trend extends to metal‐free controls: (Mg)‐N‐C contains substantially more pyridinic‐N than N‐C (Figure S5), proving Mg's capability to reconfigure nitrogen functionalities independently of Fe. Quantitative evolution of nitrogen species is summarized in Figure S6 and Table S5. Complementary high‐resolution Fe 2p spectra exhibit nearly identical Fe^2^^+^/Fe^3^^+^ signatures for both Fe(Mg)‐N‐C(1) and Fe‐N‐C (Figure S7), confirming consistent oxidation states. Notably, Mg 1s signals are undetectable in Fe(Mg)‐N‐C(1) (Figure S8), consistent with trace Mg loading (0.03 wt.%) from ICP‐OES.

High‐resolution C 1s XPS spectra of Fe(Mg)‐N‐C(1), Fe‐N‐C, (Mg)‐N‐C, and N‐C resolve four distinct components: sp^2^ carbon (284.5 eV), sp^3^ carbon (285.3 eV), C‐N/C‐O (286.4 eV), and π‐π^*^ transitions (291.3 eV) (Figure 2d,e, Figure S9) [41]. The sp^3^ signature characterizes diamond‐like carbon, while the sp^2^ component indicates ordered graphitic domains. Fe(Mg)‐N‐C(1) exhibits a significantly higher sp^2^/sp^3^ ratio (Figure S10 and Table S6), confirming enhanced graphitization and a more oxidation‐resistant carbon framework surrounding FeN_4_ sites—a critical factor for stability.

Simultaneously, the high‐resolution N 1s XPS spectra exhibit highly consistent profiles across catalysts prepared with different Mg contents in the precursors (Figures S12 and S13 and Tables S5 and S6). Meanwhile, XPS survey‐derived elemental compositions (Table S7) reveal higher nitrogen content in Fe(Mg)‐N‐C(1) relative to Fe‐N‐C, this enrichment likely increases Fe‐N_x_ site density and compensates for activity loss from reduced S1‐type sites.

Raman spectra (Figure 2f,g, Figure S16) were deconvoluted into four characteristic bands: D_1_ (≈1336 cm^−^^1^), D_2_ (≈1186 cm^−^^1^), D_3_ (≈1500 cm^−^^1^), and G (≈1592 cm^−^^1^) [42]. The D_1_ mode reflects carbon lattice defects/disorder, while the G band originates from in‐plane vibrations of sp^2^‐bonded graphitic carbon. The D_2_ band arises from edge‐plane defects near graphene boundaries, and D_3_ corresponds to amorphous sp^3^ carbon (typically anti‐correlated with G intensity). Notably lower I_D1_/I_G_ and I_D3_/I_G_ ratios for Fe(Mg)‐N‐C(1) versus Fe‐N‐C (Figure S17 and Table S8) corroborate a more ordered, oxidation‐resistant carbon architecture in the Mg‐modified catalyst.

**FIGURE 3:**
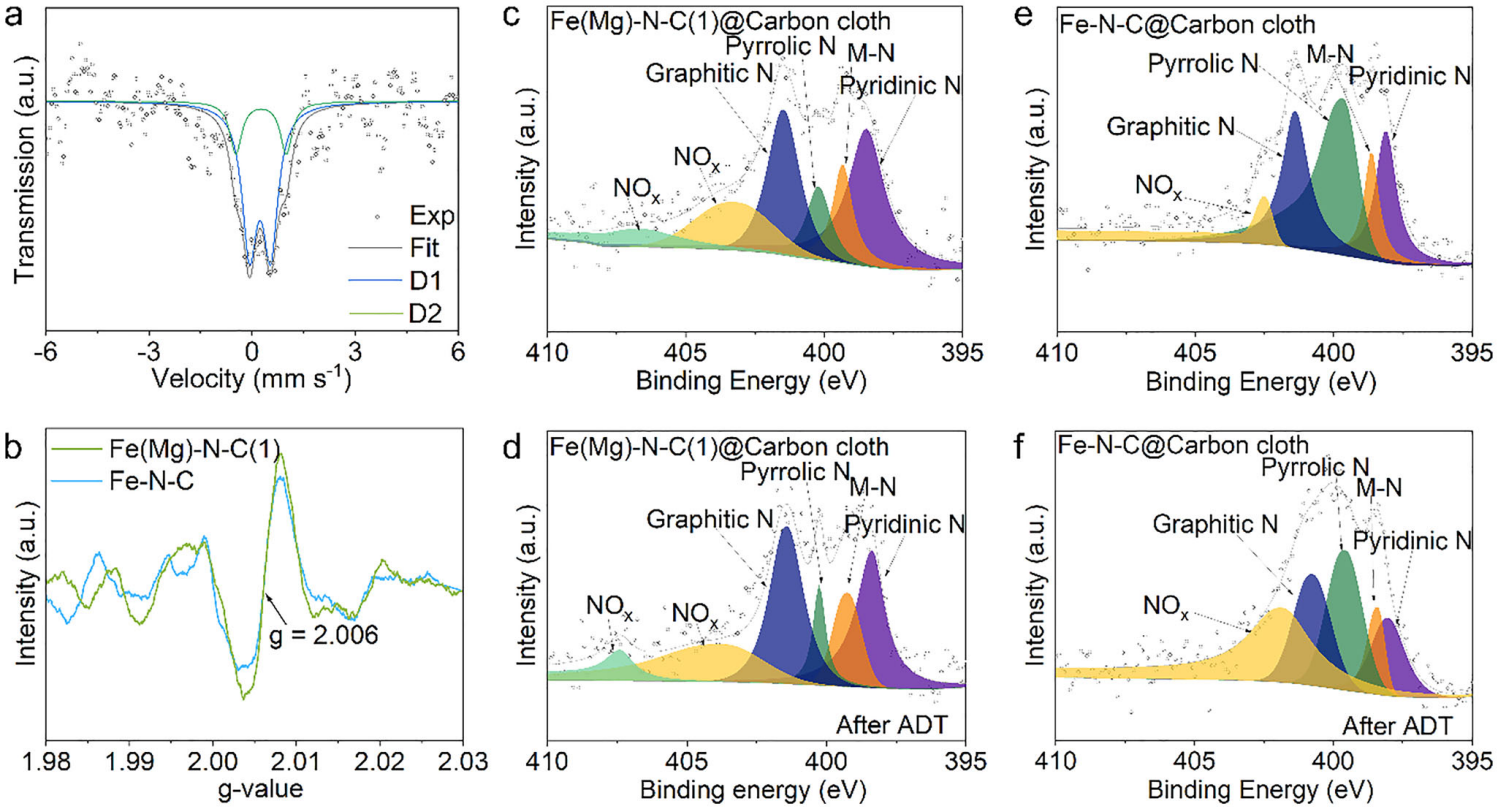
Analysis of Fe coordination environments. (a) ^57^Fe Mössbauer spectra of Fe(Mg)‐N‐C(1) at room temperature. (b) EPR spectra of Fe(Mg)‐N‐C(1) and Fe‐N‐C. (c,d) High‐resolution N 1s XPS spectra of the Fe(Mg)‐N‐C(1)@carbon cloth catalyst (c) before and (d) after ADT. (e,f) High‐resolution N 1s XPS spectra for Fe‐N‐C@carbon cloth catalysts (e) before and (f) after ADT.

To elucidate Mg's influence on Fe‐N_4_ coordination environments and electronic structures, we employed ^5^ ^7^Fe Mössbauer spectroscopy—a technique exquisitely sensitive to Fe oxidation states, coordination geometries, and electronic configurations. This provides atomic‐level insight into how Mg modifies Fe's chemical state in both catalysts. As revealed in Figures 3a and S18, Fe‐N‐C exhibits a single Mössbauer doublet (D1), while Fe(Mg)‐N‐C(1) manifests an additional doublet (D2)—unambiguous evidence of a second Fe‐N_4_ configuration. Consistent with established assignments, the doublets with quadrupole splittings (QS) of ~0.61 and ~1.47 mm s^−^^1^ are unambiguously assigned to FeN_4_C_12_ (pyrrolic‐N environment) and FeN_4_C_10_ (pyridinic‐N environment), respectively. Critically, the emergence and dominance of the lower‐QS D2 component in Fe(Mg)‐N‐C(1) demonstrate Mg‐driven partial conversion from FeN_4_C_12_ to FeN_4_C_10_, aligning precisely with XPS‐observed pyridinic‐N enrichment. Complementary EPR spectra (Figure 3b) shows significantly intensified signals for Fe(Mg)‐N‐C(1), correlating directly with elevated C/N‐vacancy concentration. These vacancies facilitate reconstruction of Fe‐N_4_ sites toward the thermodynamically stable pyridinic coordination.

Accelerated degradation tests (ADT) were conducted on carbon cloth‐supported catalysts to evaluate operational stability, monitoring ORR performance parameters and surface chemistry evolution. As evidenced by Figure S19, the Fe(Mg)‐N‐C(1) catalyst exhibits minimal degradation— with only a 6 mV shift in half‐wave potential (E_1/2_) after 5,000 cycles in 0.1 M KOH electrolyte. In stark contrast, Fe‐N‐C suffers a substantial 31 mV decay, unequivocally demonstrating the superior stability of the pyridinic‐enriched material. XPS analysis of cycled electrodes (Figure 3c–f) resolves N 1s components assigned to pyridinic‐N, Fe‐N_x_ coordination, pyrrolic‐N, graphitic‐N, and oxidized‐N species. Post‐ADT quantification reveals the Fe‐N_x_ fraction in Fe(Mg)‐N‐C(1) remains nearly unchanged (12.27 → 12.00 at.%), while Fe‐N‐C shows severe depletion (11.49 → 8.09 at.%) (Table S9, Figure S24). This divergence confirms pyridinic‐coordinated Fe‐N_4_ sites possess significantly enhanced resistance to ORR‐driven demetalization. Consistent with this mechanism, Fe‐N‐C displays pronounced proliferation of oxidized‐N species, indicating greater vulnerability of pyrrolic‐N configurations to attack by reactive oxygen intermediates during electrochemical stress. Similarly, following stability testing, XRD analysis confirmed the absence of metallic crystalline phases in the Fe(Mg)‐N‐C(1) sample. In contrast, the Fe‐N‐C sample exhibited the formation of metal oxides after ADT, indicating a reduced deactivation of Fe in the Fe(Mg)‐N‐C(1) sample (Figure S29). Furthermore, TEM images of the Fe(Mg)‐N‐C(1) sample post‐cycling reveal well‐preserved morphology, thereby demonstrating its excellent structural stability (Figure S30).

Collectively, these results establish that Mg‐driven partial reconstruction into pyridinic Fe‐N_4_ configurations simultaneously suppresses dual degradation mechanisms—demetalization and oxidative corrosion‐reinforcing active‐site integrity under accelerated operational stress.

### 2.2 Electrochemical Oxygen‐Reduction Activity and Selectivity

**FIGURE 4:**
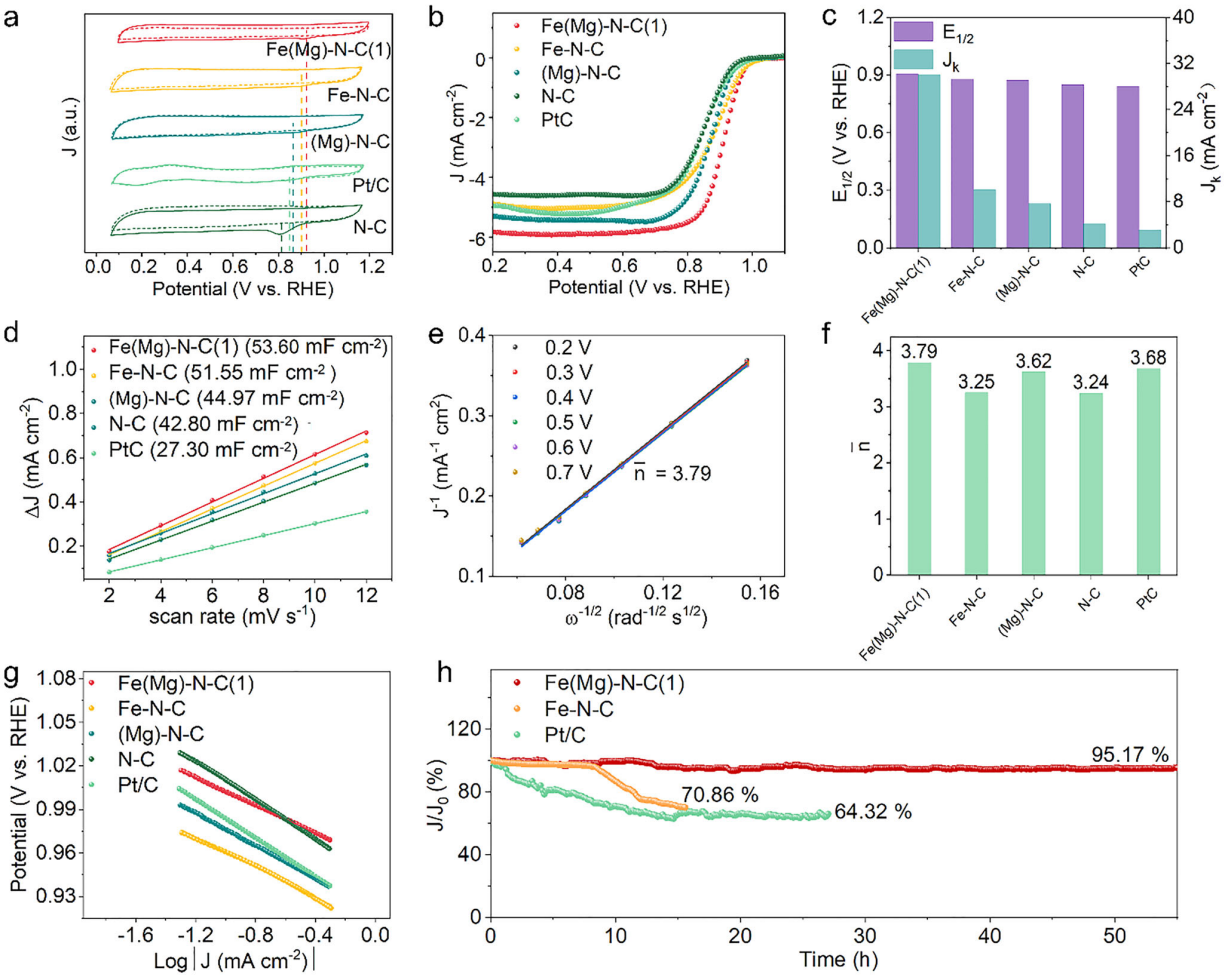
Electrocatalytic ORR performances. (a) Cyclic voltammograms for Fe(Mg)‐N‐C(1) and reference samples (Fe‐N‐C, (Mg)‐N‐C, N‐C and Pt/C) in O_2_‐saturated 0.1 M KOH at a scan rate of 50 mV s^−1^. (b) LSV curves for Fe(Mg)‐N‐C(1) and reference samples in O_2_‐saturated 0.1 M KOH at a rotation speed of 1600 rpm. (c) Values of J_k_ (at 0.85 V vs. RHE) and E_1/2_ for Fe(Mg)‐N‐C(1) and reference samples. (d) Plots of current densities as functions of scan rates. (e) The fitted K–L plots and electron transfer numbers for Fe(Mg)‐N‐C(1). (f) Values of electron transfer numbers for Fe(Mg)‐N‐C(1) and reference samples. (g) Tafel plots for Fe(Mg)‐N‐C(1) and reference samples in 0.1 m KOH. (h) Chronoamperometry data of Fe(Mg)‐N‐C(1), Fe‐N‐C, and Pt/C.

ORR performance was assessed via rotating ring‐disk electrode (RRDE) measurements in O_2_‐saturated 0.1 M KOH. Cyclic voltammetry (CV) initially revealed Fe(Mg)‐N‐C(1)’s superior activity, exhibiting the most positive reduction peak potential (Figure 4a). Linear sweep voltammetry (LSV) further resolved performance differences: Fe(Mg)‐N‐C(1) achieved a half‐wave potential (E_1/2_) of 0.91 V vs. RHE—outperforming Fe‐N‐C (0.89 V vs. RHE), (Mg)‐N‐C (0.87 V vs. RHE), N‐C (0.85 V vs. RHE), and commercial Pt/C (0.84 V vs. RHE) (Figure 4b). Critically, Fe(Mg)‐N‐C(1) delivered a kinetic current density (J_k_) of 30.1 mA cm^−^^2^ at 0.85 V, markedly exceeding counterparts: Fe‐N‐C (10.1 mA cm^−^^2^), (Mg)‐N‐C (7.8 mA cm^−^^2^), N‐C (4.2 mA cm^−^^2^), and Pt/C (3.1 mA cm^−^^2^). Summary analysis (Figure 4c) unequivocally establishes Fe(Mg)‐N‐C(1) as the leading ORR catalyst in alkaline media. This enhancement stems from Mg‐assisted nitrogen retention and site reconstruction, which preserve active Fe‐N_4_ populations while accelerating reaction kinetics.

Electrochemically active surface area (ECSA) was determined from cyclic voltammograms at varied scan rates. As evidenced by Figure 4d and Figure S31, Fe(Mg)‐N‐C(1) exhibits the highest double‐layer capacitance (C_dl_) of 53.6 mF cm^−^^2^—surpassing Fe‐N‐C (51.6 mF cm^−^^2^), (Mg)‐N‐C (45.0 mF cm^−^^2^), and N‐C (42.8 mF cm^−^^2^)—confirming its superior density of accessible active sites. Rotation‐dependent LSV profiles for Fe(Mg)‐N‐C(1) and reference catalysts are presented in Figure 4e and Figures S32 and S33. Koutecký‐Levich (K‐L) analysis yields an electron transfer number (*n*) of 3.79 (0.2–0.7 V vs. RHE) for Fe(Mg)‐N‐C(1) (Figure 4f), approaching the ideal four‐electron pathway and exceeding all reference materials. Complementary kinetic assessment reveals Fe(Mg)‐N‐C(1) achieves the lowest Tafel slope (47.7 mV dec^−^^1^) among tested catalysts‐including Fe‐N‐C, (Mg)‐N‐C, N‐C, and Pt/C (Figure 4g, Table S10)—demonstrating accelerated ORR kinetics. Collectively, these metrics establish Fe(Mg)‐N‐C(1) as delivering exceptional overall ORR performance in alkaline media, attributable to Mg‐assisted site reconstruction optimizing both active‐site exposure and reaction dynamics.

Operational stability was further evaluated via chronoamperometry at 0.46 V vs. RHE in O_2_‐saturated 0.1 m KOH (1600 rpm, Figure 4h). Following 55 h of continuous operation, Fe(Mg)‐N‐C(1) retained 95.2% of its initial current density—a stark contrast to Fe‐N‐C and Pt/C, which exhibited rapid current decay. This exceptional stability originates from Mg‐induced conversion of unstable S1 sites (pyrrolic‐N‐coordinated FeN_4_C_12_) into robust S2 configurations (pyridinic‐N‐coordinated FeN_4_C_10_), reinforcing structural integrity against demetalization and oxidative degradation under operational conditions.

In addition, LSV curves of these samples were evaluated in an acidic electrolyte. The half‐wave potential of Fe(Mg)‐N‐C (0.764 V vs. RHE) was lower than that of Pt/C (0.848 V vs. RHE) but marginally higher than that of Fe‐N‐C (0.73 V vs. RHE) (Figure S34). After 5,000 CV cycles, the Fe(Mg)‐N‐C catalyst exhibited a half‐wave potential decay of only 16 mV, which is significantly lower than that of Fe‐N‐C (74 mV), indicating markedly improved durability in acidic electrolyte (Figure S35).

Figure S36 provides supporting information by presenting LSV curves of samples with varying Mg content in the precursors. The nitrogen‐fixation effect of Mg contributes to a slight enhancement in catalytic activity, which compensates for the activity loss caused by the depletion of S1 sites. Consequently, there exists an optimal magnesium addition level that balances the catalyst's activity, preventing excessive performance degradation due to Mg incorporation.

Furthermore, to assess the generalizability of the strategy, other lightweight metals (Li, Al, K) were incorporated into Fe‐N‐C materials. After 5,000 CV cycles, both lightweight‐metal‐ modified catalysts exhibited negligible half‐wave potentials decay compared to that of Fe‐N‐C, as evidenced by the decreases from 0.889 to 0.881 V (vs. RHE) for Fe(Li)‐N‐C, from 0.867 to 0.865 V (vs. RHE) for Fe(Al)‐N‐C, and from 0.876 to 0.869 V (vs. RHE) for Fe(K)‐N‐C (Figure S37). These results demonstrate the broad applicability of the proposed approach.

### 2.3 Performance in Zinc‐Air Batteries Using Fe(Mg)‐N‐C(1)

**FIGURE 5:**
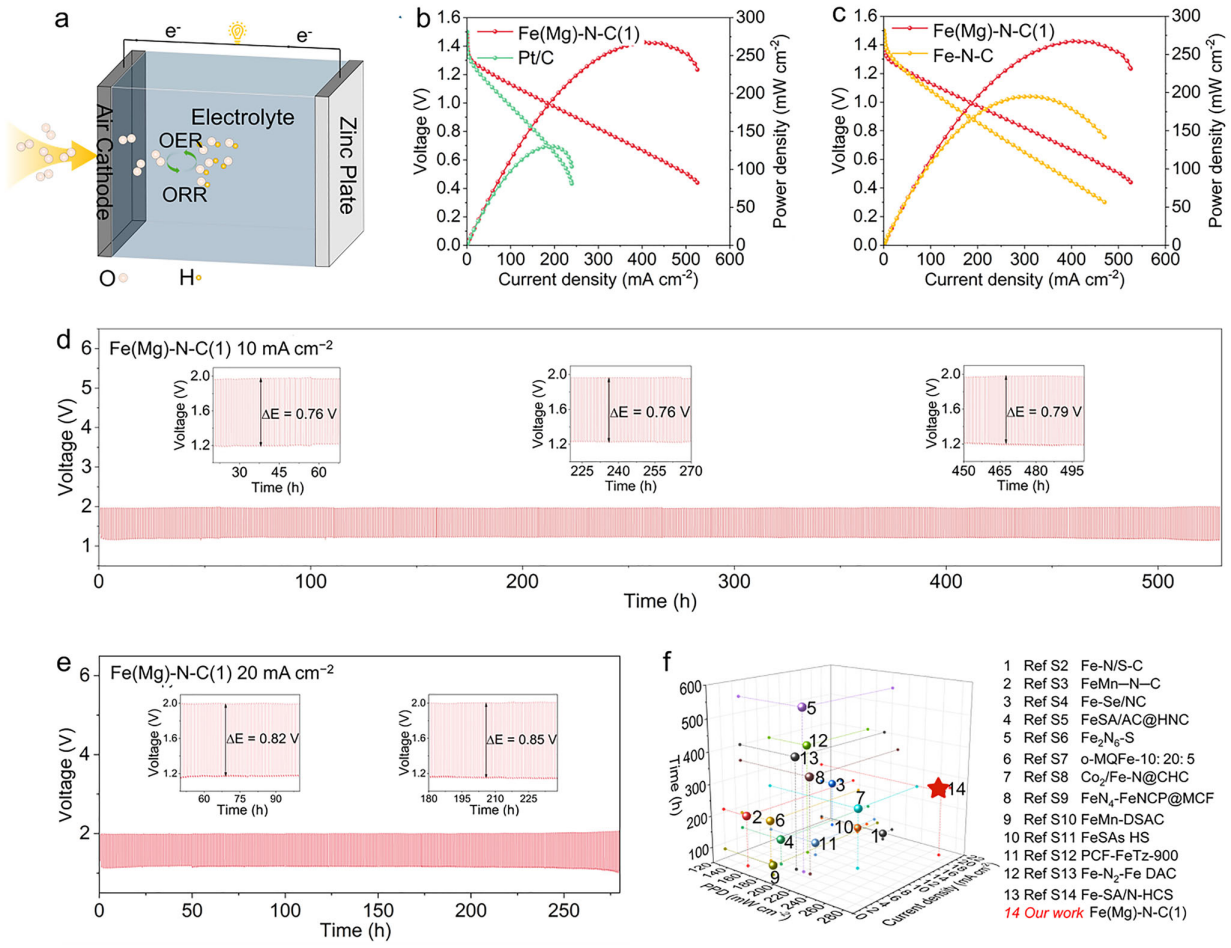
ZAB performance. (a) ZAB structure schematic. (b,c) Discharge polarization curves and corresponding power density curves. (d,e) Cycling performance of ZABs with Fe(Mg)‐N‐C(1) cathode at a current density of (d) 10 mA cm^−2^ and (e) 20 mA cm^−2^. (h) Comparison with reported ZABs. The values of coordinates (m, n, o) in the graph represent the current density, cycle time, and power densities, respectively.

To evaluate practical utility, zinc‐air batteries (ZABs) were fabricated with Fe(Mg)‐N‐C(1) air cathodes and zinc foil anodes (Figure 5a). Polarization and power‐density profiles (Figure 5b) reveal Fe(Mg)‐N‐C(1) achieves a record peak power density of 271.4 mW cm^−^^2^ at 407.2 mA cm^−^^2^‐significantly surpassing Pt/C (130.2 mW cm^−^^2^ at 199.3 mA cm^−^^2^) and Fe‐N‐C (194.9 mW cm^−^^2^ at 313.8 mA cm^−^^2^). The Fe(Mg)‐N‐C(1) based ZAB maintains a stable open‐circuit voltage of 1.482 V (Figure S38) and delivers a specific capacity of 809.9 mAh g^−^^1^ (Figure S39), confirming exceptional electrochemical performance.

Critically, Fe(Mg)‐N‐C(1) enables ZAB operation exceeding 530 h at 10 mA cm^−^^2^ (Figure 5d), with minimal voltage polarization growth (initial charge/discharge gap: 0.76 V → 0.79 V after 530 h). Stability persists for 260 h even at 20 mA cm^−^^2^ (Figure 5e), while Fe‐N‐C counterparts fail after only ~100 h (10 mA cm^−^^2^) and ~65 h (20 mA cm^−^^2^) (Figure S40). As benchmarked in Figure 5f and Table S11, Fe(Mg)‐N‐C(1) delivers unprecedented combination of high power density and durability, outperforming most reported Fe‐based cathodes. Collectively, these results establish that Mg incorporation simultaneously boosts ZAB performance and extends device longevity through two synergistic effects: (1). S1→S2 site transformation (pyrrolic‐N → pyridinic‐N coordination) enhancing structural robustness. (2). Optimized nitrogen retention increasing active Fe‐N_4_ site density to elevate catalytic activity.

## 3 Conclusion

We demonstrate a Mg‐driven coordination reconstruction strategy that transforms unstable pyrrolic Fe‐N_4_ sites (S1) into robust pyridinic configurations (S2), yielding a highly active and durable Fe‐N‐C catalyst (Fe(Mg)‐N‐C(1)). This targeted reconfiguration—validated by ^5^ ^7^Fe Mössbauer spectroscopy, XPS, and Raman analyses—enhances carbon matrix robustness through increased graphitization while suppressing dual degradation pathways (Fe demetalization and carbon oxidation) under ORR conditions. The optimized catalyst achieves exceptional ORR performance with a half‐wave potential (E_1/2_) of 0.91 V and 95.2% current retention after 55 h operation, substantially outperforming Pt/C and conventional Fe‐N‐C. As a zinc‐air battery cathode, it delivers a record peak power density (271.4 mW cm^−^^2^) and unprecedented operational longevity (>530 h at 10 mA cm^−^^2^). This work establishes Mg incorporation as a generalizable design principle for precise coordination‐environment engineering, resolving the persistent activity‐stability trade‐off in single‐atom catalysts. Our sacrificial templating approach provides a scalable pathway to develop next‐generation Fe‐based electrocatalysts that unite precious‐metal competitiveness with industrial‐grade durability for sustainable energy applications.

## Conflicts of Interest

The authors declare no conflict of interest.

## Supporting information




**Supporting File**: smll72673‐sup‐0001‐SuppMat.docx.

## Data Availability

The data that support the findings of this study are available from the corresponding author upon reasonable request.
