# Supplementary material for: Breaking the ORR Trade‐Off via Mg‐Steered Fe‐N4 Pyridinic Conversion
Source: Small. 2026 Feb 4;22(19):e14722. doi: 10.1002/smll.202514722 (PMC13040119; doi:10.1002/smll.202514722)
Supplement: Supplementary file 1 — Supporting File: smll72673‐sup‐0001‐SuppMat.docx. [file SMLL-22-e14722-s001.docx]

**Breaking the ORR Trade-off via Mg-Steered Fe-N₄ Pyridinic Conversion**

Si-Qi Sun, ^1#^ Ya-Peng Cheng, ^2,3#^ Hai-Ning Zhang,^1,4^ Jia-Min Lyu,^1^ Xiao-Yun Li,^5^ Ke Lyu,^1^ Ming-Hui Sun,^1*^ Shen Yu,^1^ Wen-Mao Tu,^1,4^ Andreu Cabot,^2,6*^ Li-Hua Chen^1*^

^1^ State Key Laboratory of Advanced Technology for Materials Synthesis and Processing, Wuhan University of Technology, No. 122 Luoshi Road, Wuhan 430070, China

^2^ Catalonia Institute for Energy Research-IREC, Sant Adrià de Besòs, 08930, Catalonia, Spain

^3^ Universitat de Barcelona, Barcelona, 08028, Catalonia, Spain

^4^ Hubei Key Laboratory of Fuel Cell, Wuhan University of Technology, No. 122 Luoshi Road, Wuhan 430070, China

^5^ State Key Laboratory of Silicate Materials for Architectures, Wuhan University of Technology, 122 Luoshi Road, Wuhan 430070, P. R. China.

^6^ ICREA, Pg. Lluís Companys 23, Barcelona, 08010, Catalonia, Spain

^#^ These authors contributed equally to this study.

^*^Corresponding authors:

Ming-Hui Sun ([sunminghui@whut.edu.cn](mailto:sunminghui@whut.edu.cn))

Andreu Cabot ([acabot@irec.cat](mailto:acabot@irec.cat))

Li-Hua Chen (chenlihua@whut.edu.cn)

**Experimental Methods**

**1. Chemicals and Materials**

Zn(COOH)_2_·2H_2_O (99%), N, N-dimethylformamide (DMF, AR), anhydrous methanol (MeOH, AR), and anhydrous ethanol (EtOH, AR) were supplied by Beijing Tongguang Fine Chemical Co., Ltd. FeCl_2_ (99%), Mg (COOH)_2_·4H_2_O (99%), Adenine (AD, 99%), terephthalic acid (H_2_BDC, 99%), ammonium chloride (NH_4_Cl, AR), potassium hydroxide (KOH, 85%) and perchloric acid (HClO_4_, 99%) were purchased from Shanghai Aladdin Biochemical Technology Co., Ltd. The Nafion solution (5 wt. %) and the commercial 20 wt. % Pt/C catalyst were received from InnoChem and Shanghai Hesen Electric Co., Ltd, respectively. RuO_2_ (99.95%) was supplied by Alfa Aesar. All chemicals and solvents were used without further purification. Ultrapure water was used throughout the experiment.

**2. Preparation of IISERP-MOF27**

The synthesis of IISERP-MOF27 was based on the reported literature with some modifications.^1^ Typically, Zn(COOH)_2_·2H_2_O (0.266 g), H_2_BDC (0.100 g), and AD (0.162 g) were dispersed in the mixture of DMF (15 mL), H_2_O (15 mL), and MeOH (9mL) in a Teflon reaction kettle. After ultrasonic dispersion for 0.5 h, the above dispersion was placed in the autoclave and heated at 120 ℃ for 48 h. The obtained IISERP-MOF27 was washed with DMF and MeOH three times by centrifugation, dried at 80 ℃ for 12 h, and then activated at 120 ℃ for 12 h in the vacuum oven.

**3. Preparation of M-IISERP-MOF27 (M= Fe, Mg)**

During the synthesis of IISERP-MOF27, metal precursors (0.811 mg of FeCl_2_ and a certain proportion of Mg(COOH)_2_·4H_2_O) were concurrently introduced. The product named FeMg- IISERP-MOF27 (*x*) was dried overnight in an oven at 80 ℃, the variable *x* denotes the molar ratio of n(Mg) to n(Fe) in the metal precursor, which are 0.2, 0.5, 1, 1.5, and 2, respectively. Fe-IISERP-MOF27 and Mg-IISERP-MOF27 were also synthesized without adding Mg(COOH)_2_·4H_2_O and FeCl_2_, respectively.

**4. Preparation of Fe(Mg)-N-C (*x*), Fe-N-C, (Mg)-N-C, N-C catalysts**

For Fe(Mg)-N-C (*x*), Fe-N-C, (Mg)-N-C, N-C catalysts, the obtained 0.1 g FeMg-IISERP-MOF27 (*x*), Fe-IISERP-MOF27, Mg-IISERP-MOF27, IISERP-MOF27 precursor, respectively, was placed in a quartz boat and 1.0 g of NH_4_Cl at the intake, then heated at 900 ℃ for 2 h in a tube furnace with a heating rate of 5 °C min^-1^ under flowing Ar atmosphere.

**5. Material Characterization**

X-ray diffraction (XRD) was measured on the Bruker D8 ADVANCE with Cu Kα (λ = 1.54178 Å) radiation. Scanning electron microscope (SEM) images were taken on TESCAN MIRA LMS. The transmission electron microscopy (TEM) images and energy-dispersive X-ray spectroscopy (EDX) were collected on FEI Talos F200X, and the samples were prepared on a copper mesh after ultrasonication. The N_2_ adsorption/desorption curves were tested at 77 K using a Micromeritics 3Flex surface area analyzer. The metal content was determined using inductively coupled plasma optical emission spectrometry (ICP-OES), which was carried out on an Agilent 700 system. Powder X-ray photoelectron spectroscopy (XPS) analysis used Al Kα as an exciting radiation source on an Escalab 250Xi system. The Raman spectra were taken on a Renishaw in LabRAM Odyssey spectrometer system. The EPR spectra were collected from a pulsed electron paramagnetic resonance spectrometer (Bruker EMXplus-6/1). The Mössbauer measurements were performed at room temperature using a conventional spectrometer (Germany, Wissel MS-500) in transmission geometry with constant acceleration mode. A ^57^Co (Rh) source with an activity of 25 mCi was used. The velocity calibration was done with a room temperature α-Fe absorber. The spectra were fitted by the software Recoil using Lorentzian Multiplet Analysis.

**6. Electrode Preparation and Electrochemistry Test**

All the electrochemical measurements were performed in a three-electrode system controlled by the CHI 760 electrochemical station (Shanghai Chenhua, China). A glassy carbon (GC) round disk electrode (RDE) (5 mm in disk diameter) served as the working electrode. An Ag/AgCl (saturated KCl solution) electrode and a platinum electrode were used as the reference and counter electrode in 0.1 M KOH electrolyte, respectively. A saturated calomel electrode was employed as the reference electrode in a 0.1 M HClO_4_ electrolyte solution. To prepare the catalyst ink, 5 mg of the catalyst was mixed with 1 mL of a solution containing 700 μL of ethanol, 280 μL of deionized aqueous solution, and 20 μL of Nafion solution (5 wt. %) under ultrasonication for 1 h. Then, 12 µL of the catalyst ink was pipetted to the surface of the pre-polished glassy carbon electrode, with the catalyst loading controlled at 0.3 mg·cm^-1^. The ink was dried in air at room temperature. Pt/C catalyst (20 wt. %) was also prepared under the same conditions. All the tests were conducted in 0.1 M KOH or HClO_4_ electrolyte that bubbled with O_2_ or N_2_ for 30 min before the measurement. Cyclic voltammograms (CVs) curves ranging from 1.2 to 0 V (vs. RHE) at a sweep rate of 50 mV s^-1^ were used for the initial activation of all catalysts. Linear sweep voltammetry (LSV) curves were obtained to investigate the performance of the catalysts at a scan rate of 5 mV s^−1^ with a rotating speed of 1600 rpm. The LSV curves at the same scan rate were collected in an N_2_-saturated atmosphere to eliminate the interference of background currents. All potentials are converted to RHE by the Nernst equation:

*E_RHE_ = E_(Ag/AgCl)_ +E_0(Ag/AgCl)_ +* 0.059 * *PH* (1)

where the *E_(Ag/AgCl)_* is the applied potential in the experiment, the *pH* value of 0.1 M KOH electrolyte is 13 and *E 0(Ag/AgCl_)_* is 0.199 V.

*E_RHE_ = E_(SCE)_ +E_0(SCE)_ +* 0.059 * *PH* (2)

where the *E_(SCE)_* is the applied potential in the experiment, the *pH* value of 0.1 M HClO_4_ electrolyte is 1 and *E 0(Ag/AgCl_)_* is 0.241 V.

The kinetic current density of the reaction is calculated as follows:

$J_{k}=\frac{J*J_{L}}{J_{L}-J}$ (3)

Where *J_K_*, *J_L_*, and *J* denote the kinetic current density, diffusion-limiting current density, and current density, respectively.

The electrochemical double-layer capacitance (*C_dl_*) value was determined by the CV curves obtained at different scan rates in the non-Faraday zone, which can be calculated by the equation:

$C_{dl}=\frac{j_{a}-j_{c}}{2\times v}$ (4)

where *j_a_* and *j_c_* is the anodic and cathodic current density, respectively, recorded at the middle (1.07 V vs. RHE) of the selected potential range, and *v* is the scan rate.

The average number of electrons transferred (*n*) per oxygen molecule determined from rotating disk electrode (RDE) was calculated according to the following Koutechy Levich (K-L) equation:

1/*J* = 1/*J_K_* +1/(*Bω^1/2^*)

*B* = 0.2*nFC_0_D_0_*^2/3^*v*^-1/6^

*J_K_* = *nFkC_0_* (5)

Where *J* and *J_K_* are the measurements of current density and dynamic current density, respectively; *ω* is the rotational speed; *n* is the number of transferred electrons; *F* is the Faraday constant (96485 C mol^-1^); *C_0_* is the saturated concentration of O_2_ in the electrolyte (1.21×10^-6^ mol cm^-3^); *D_0_* is the diffusion coefficient of O_2_ (1.9×10^-5^ cm^-2^ s^-1^); *μ* is the kinetic viscosity of the electrolyte (0.01 cm^2^ s^-1^); *k* is the electron transfer rate constant. Tafel slopes were also determined from the K-L equation. Accelerated durability test (ADT) involves evaluating the changes of the LSV curves (1600 rpm) before and after performing 5,000 cycles of CV ranging from 0.74 to 1.05 V (vs. RHE) at a sweep rate of 100 mV s^-1^ in O_2_-saturated 0.1 M KOH electrolyte.

**7. Zinc-Air Battery assembly and test**

Fe(Mg)-N-C(1), Fe-N-C, or Pt/C was used as the air cathode and a zinc plate as the anode. The mass loading of the total catalyst onto carbon paper was 1.0 mg cm^−2^. 6.0 M KOH solution containing 0.2 M zinc acetate was served as electrolyte. The polarization curve measurements were performed by LSV (5 mV/s) with a CHI760e electrochemical working station. The current density and power density were normalized to the effective surface area of air electrode. The open-circuit voltage test was conducted to test the battery voltage in the open-circuit state. The test was kept for 1 hour to observe the stability of the voltage over time. The specific capacity was calculated according to the equation below:

$Specific capacity=\frac{\mathrm{current}*service hours}{weight of consumed zinc}$ (6)

The galvanostatic discharge/charge cycles of ZABs lasted for 1 hour (0.5 hours of discharge followed by 0.5 hours of discharge) to detect durability.

**Supplement Figures**

**
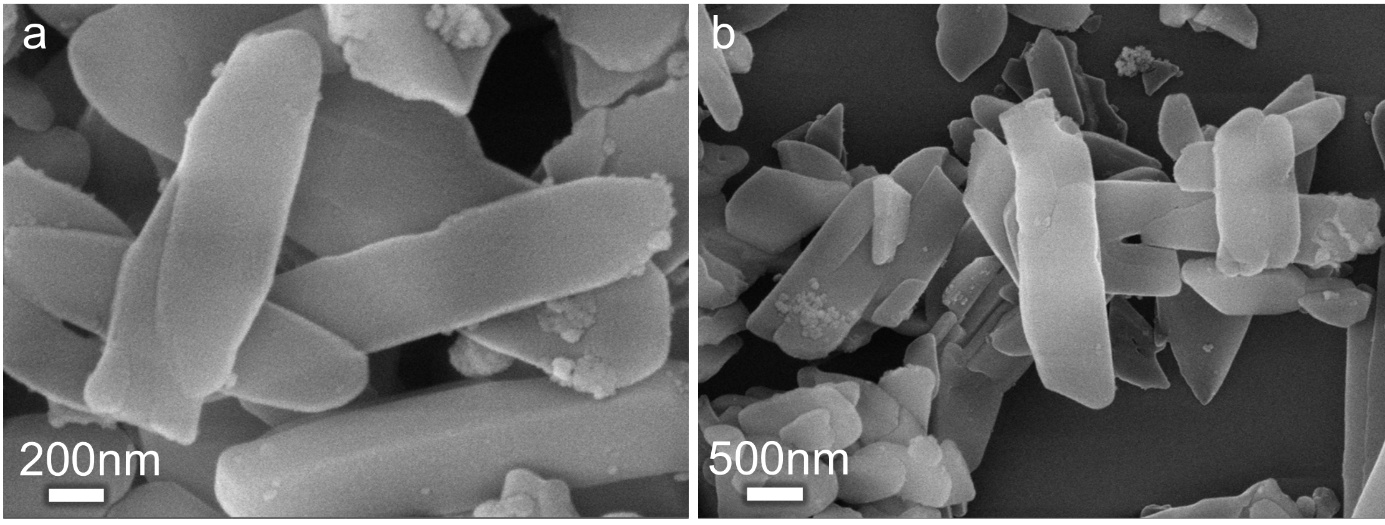
**

**Figure S1.** (a-b) SEM images of the Fe(Mg)-N-C(1) precursor.


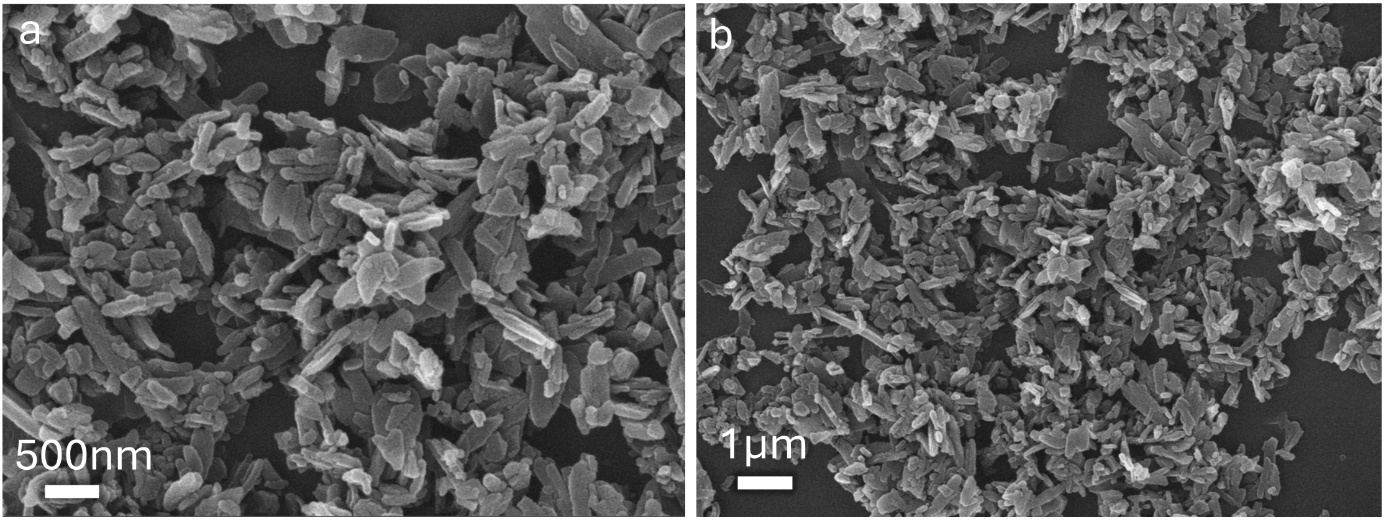


**Figure S2.** (a-b) SEM images of Fe(Mg)-N-C(1).


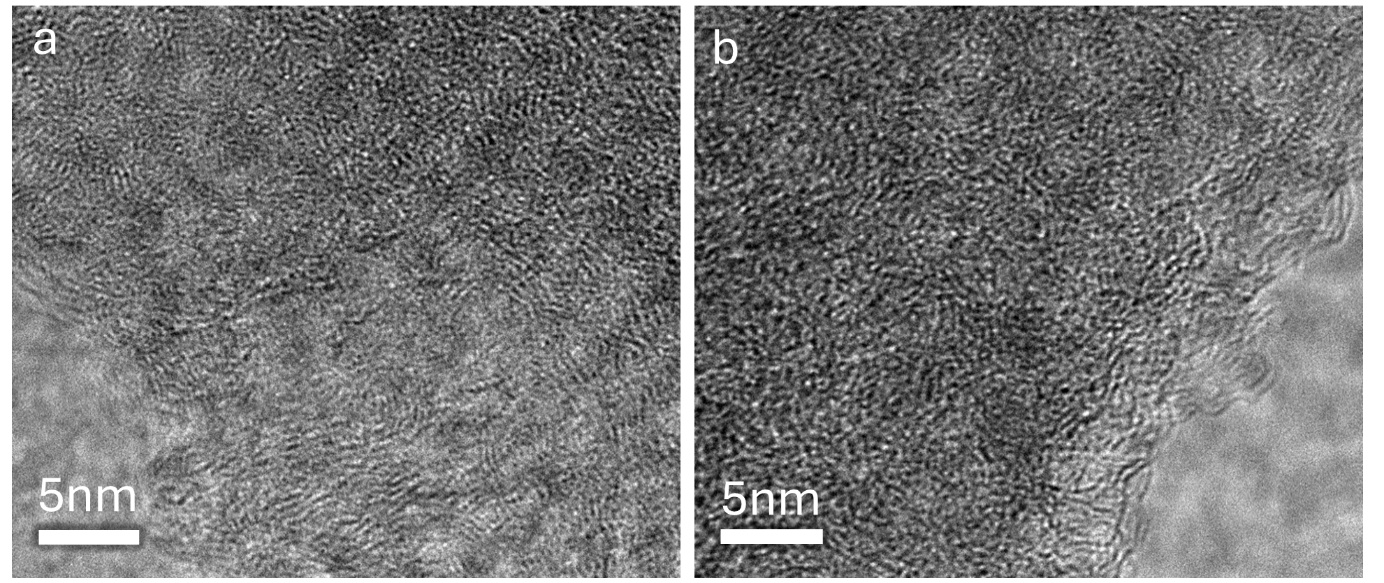


**Figure S3.** (a-b) TEM images of Fe(Mg)-N-C(1).


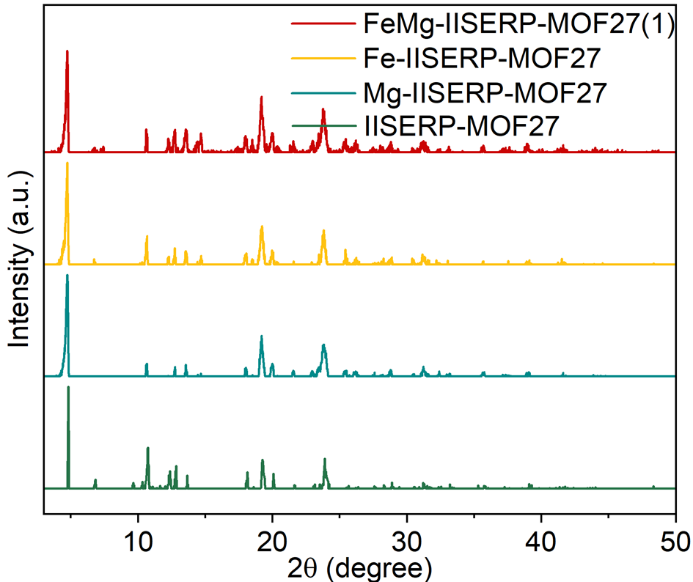


**Figure S4.** XRD patterns of FeMg-IISERP-MOF27(1), Fe-IISERP-MOF27, Mg-IISERP-MOF27, and IISERP-MOF27.


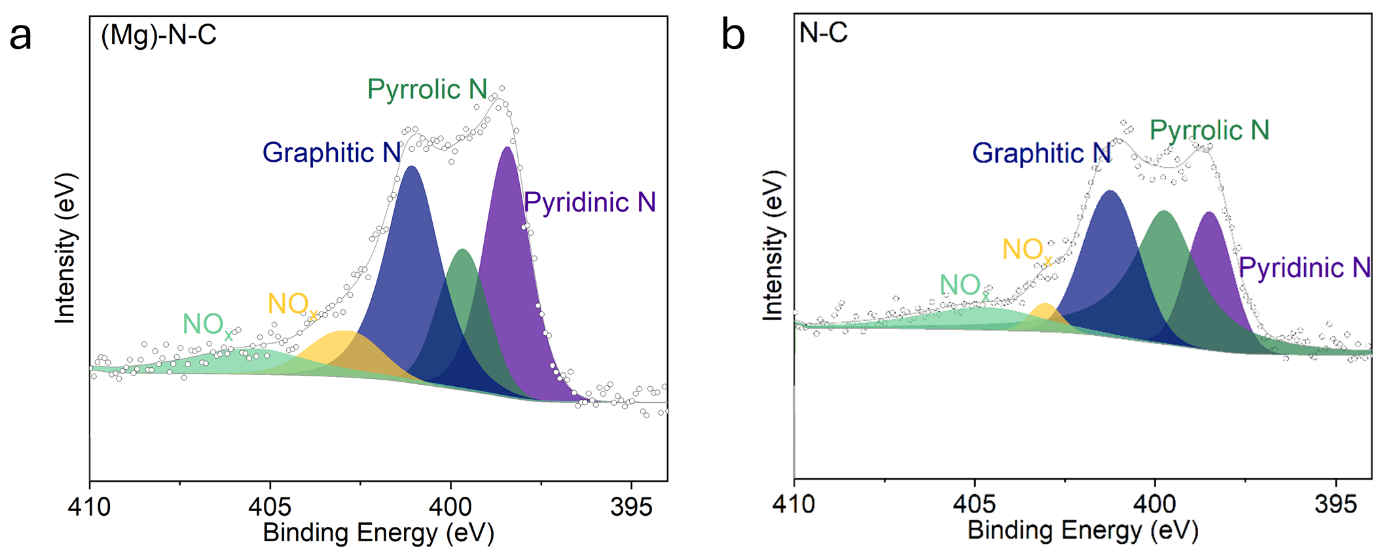


**Figure S5.** (a, b) High-resolution N 1s XPS spectra of (a) (Mg)-N-C and (b) N-C.


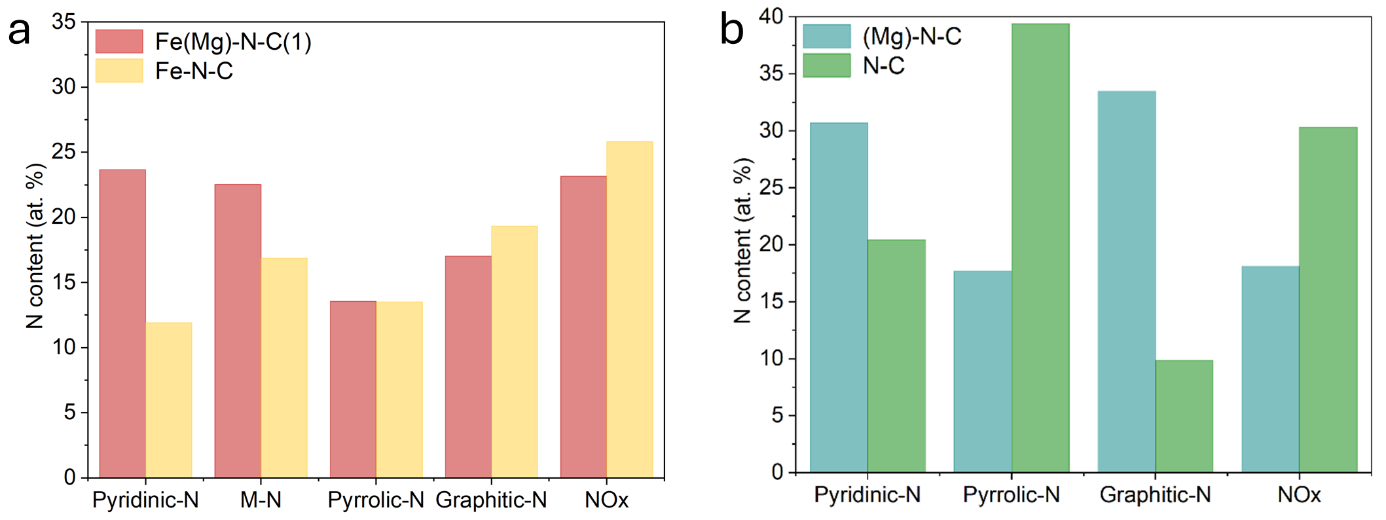


**Figure S6.** (a, b) Surface compositions (at. %) of (a) Fe(Mg)-N-C(1) and Fe-N-C and (b) (Mg)-N-C and N-C as obtained from the N 1s XPS spectra.


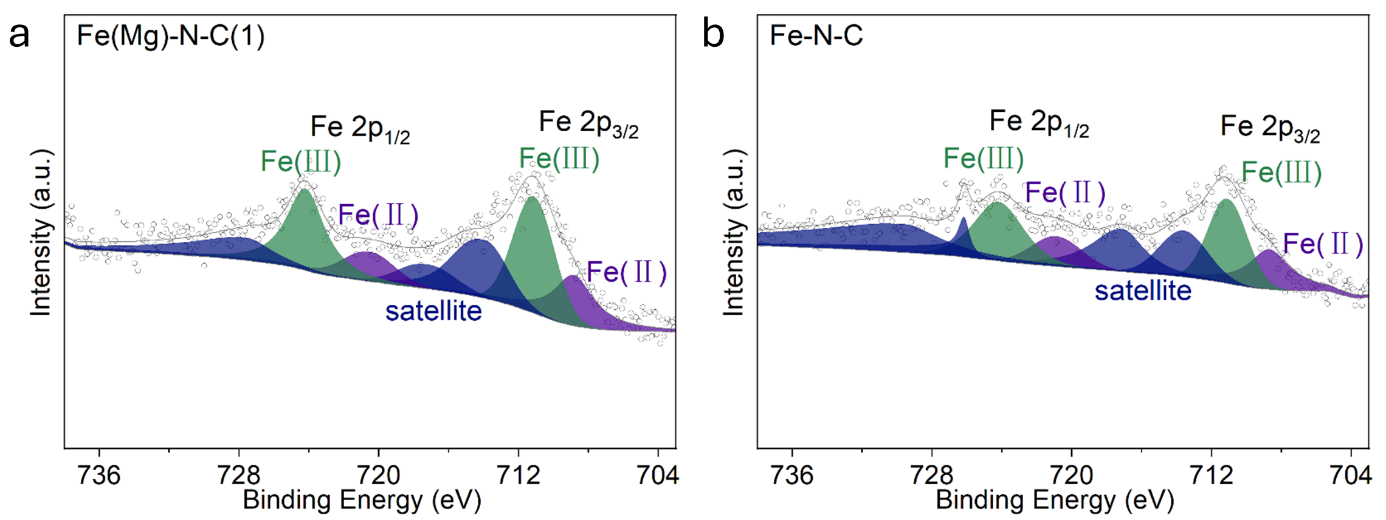


**Figure S7.** (a, b) High-resolution Fe 2p XPS spectra of (a) Fe(Mg)-N-C(1) and (b) Fe-N-C.


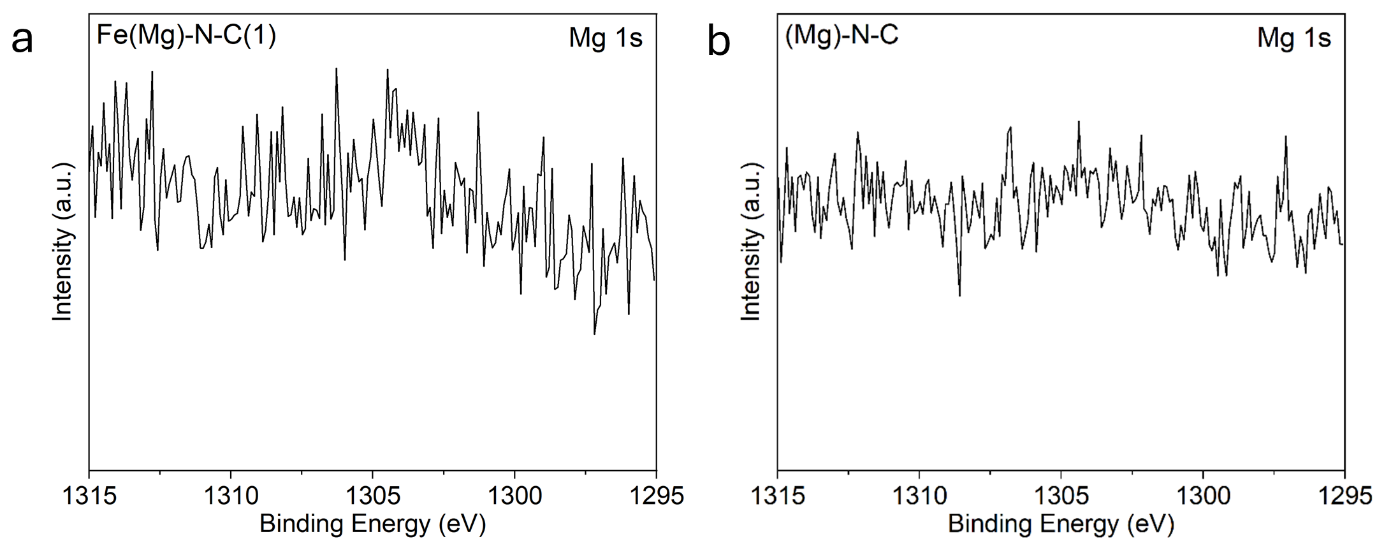


**Figure S8.** (a, b) High-resolution Mg 1s XPS spectra of (a) Fe(Mg)-N-C(1) and (b) (Mg)-N-C.


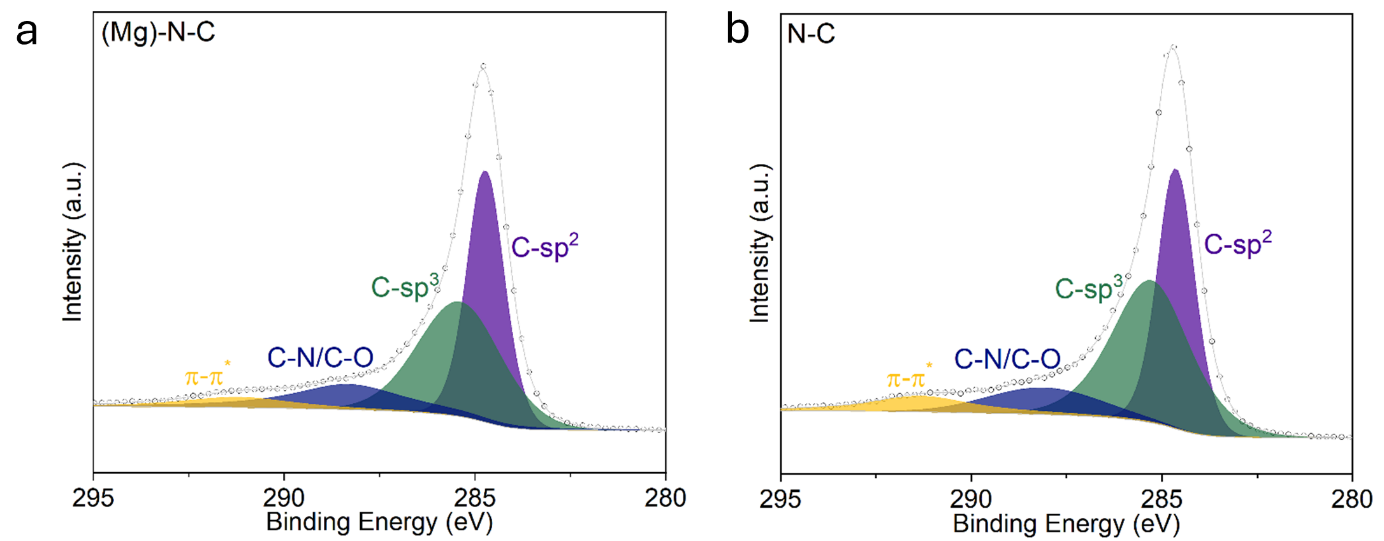


**Figure S9.** (a, b) High-resolution C 1s XPS spectra of (a) (Mg)-N-C and (b) N-C.


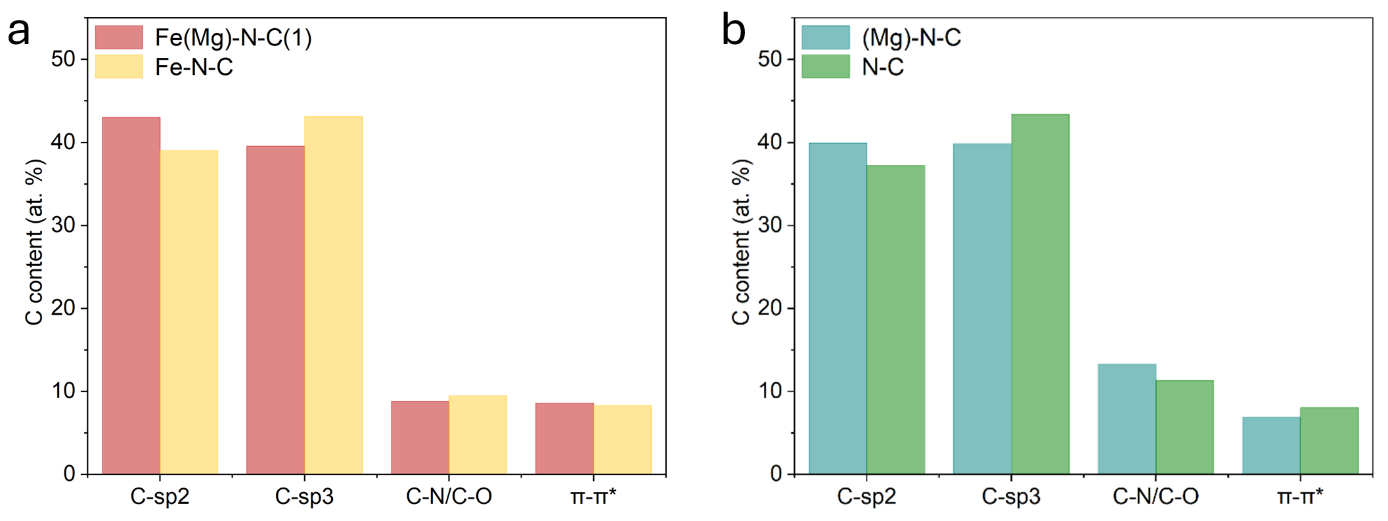


**Figure S10.** (a, b) Carbon surface compositions (at. %) of (a) Fe(Mg)-N-C(1) and Fe-N-C, and (b) (Mg)-N-C and N-C as obtained from the C 1s XPS spectra.


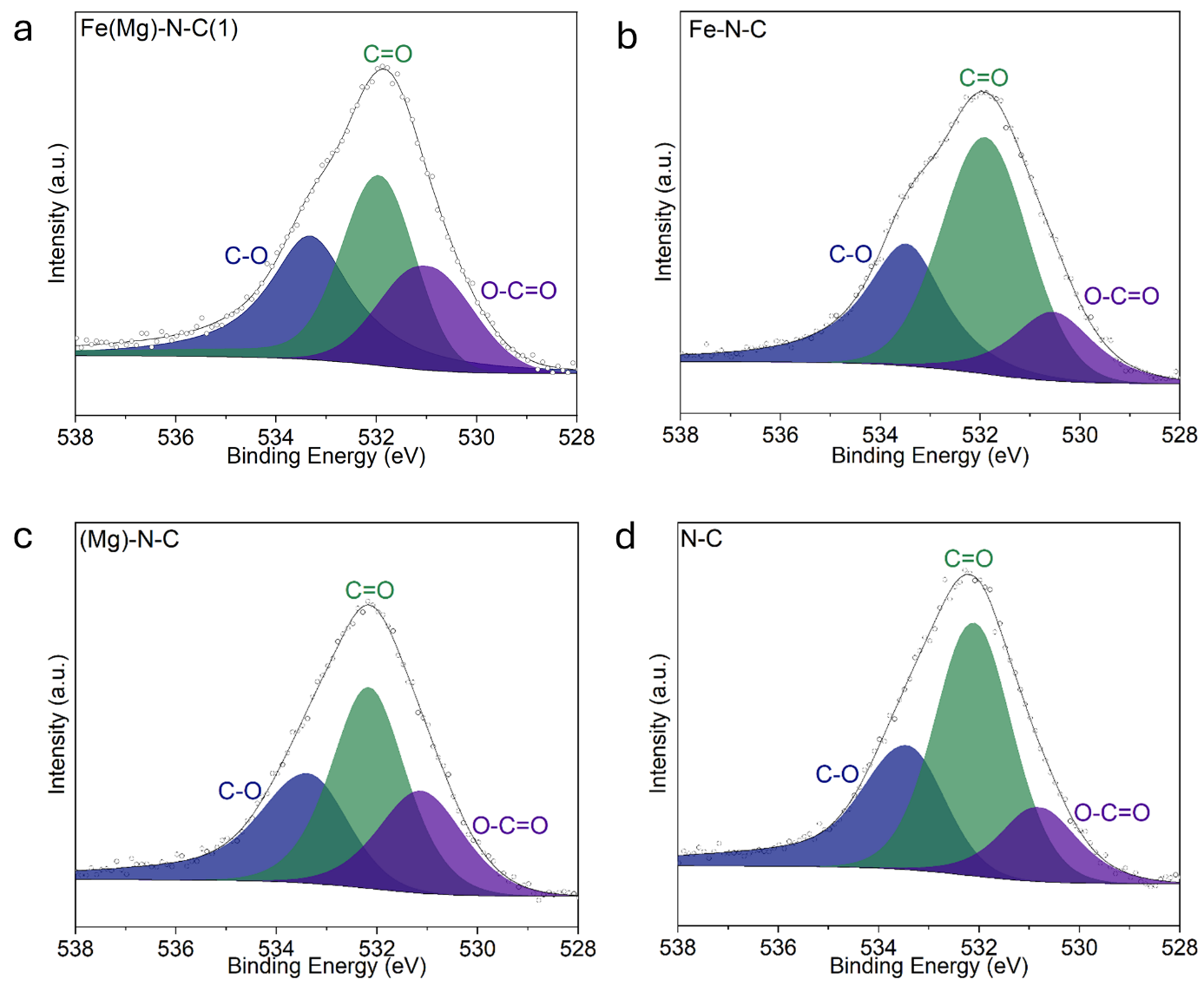


**Figure S11.** (a-d) High-resolution O 1s XPS spectra of (a) Fe(Mg)-N-C(1), (b) Fe-N-C, (c) (Mg)-N-C, and (d) N-C.


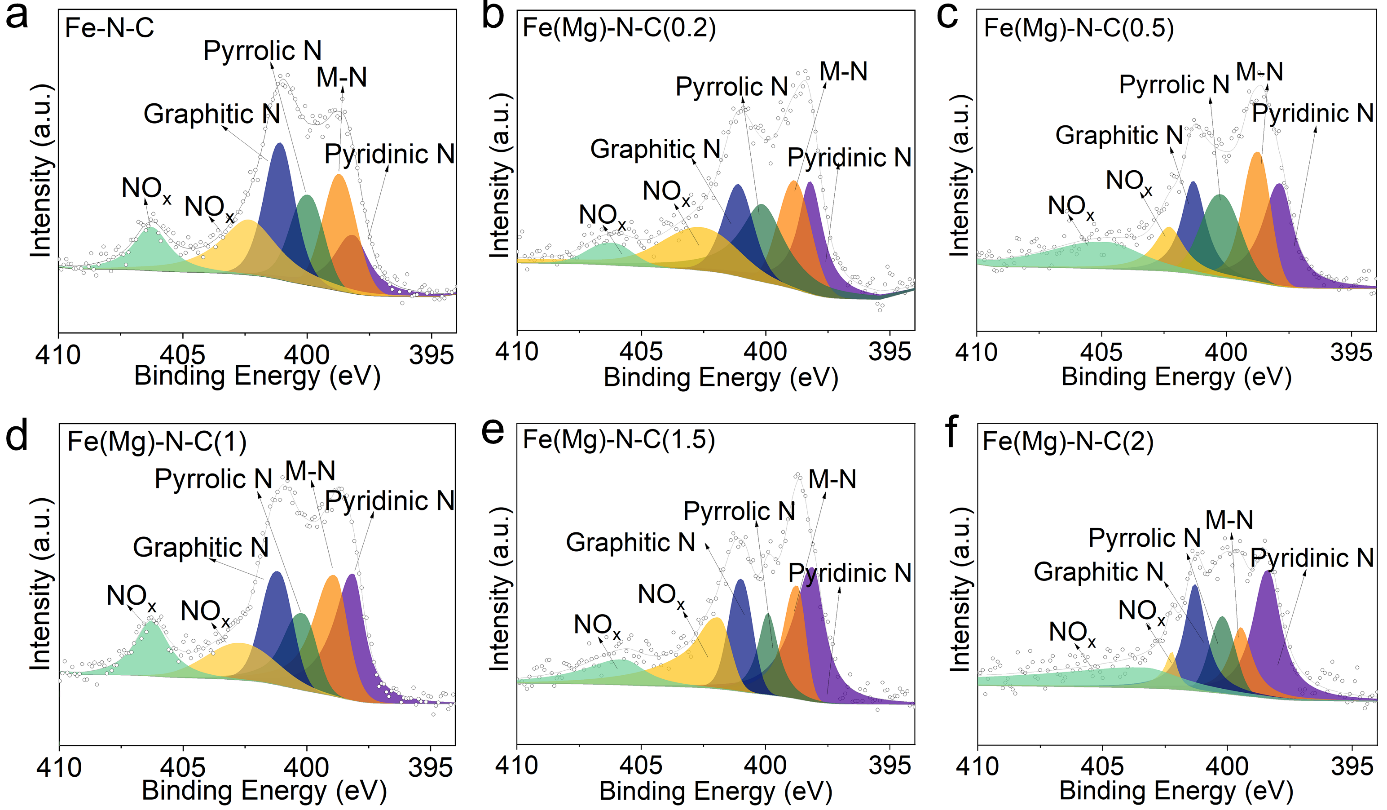


**Figure S12.** (a-f) High-resolution N 1s XPS spectra of samples with varying Mg content in the precursor.


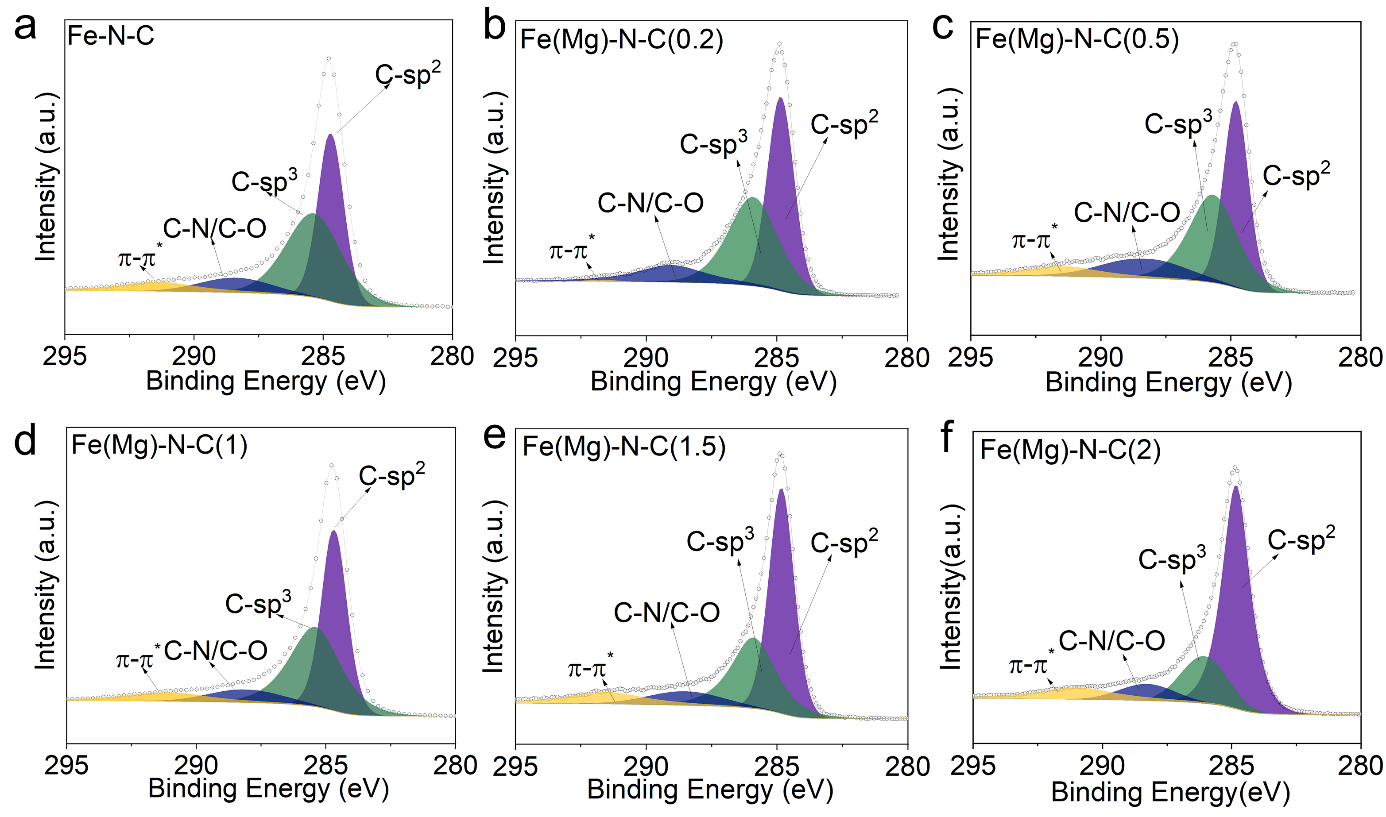


**Figure S13.** (a-f) High-resolution C 1s XPS spectra of samples with varying Mg content in the precursor.


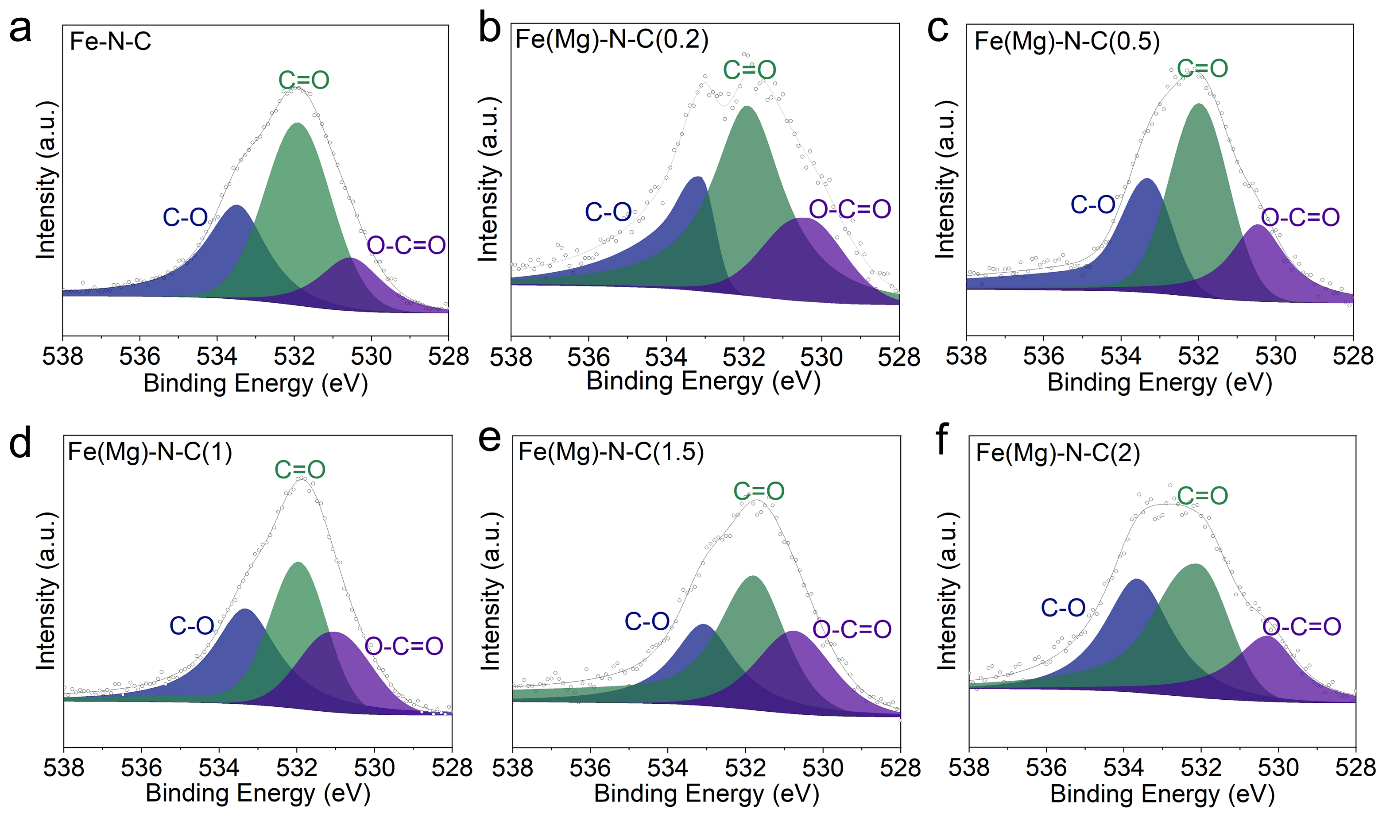


**Figure S14.** (a-f) High-resolution C 1s XPS spectra of samples with varying Mg content in the precursor.


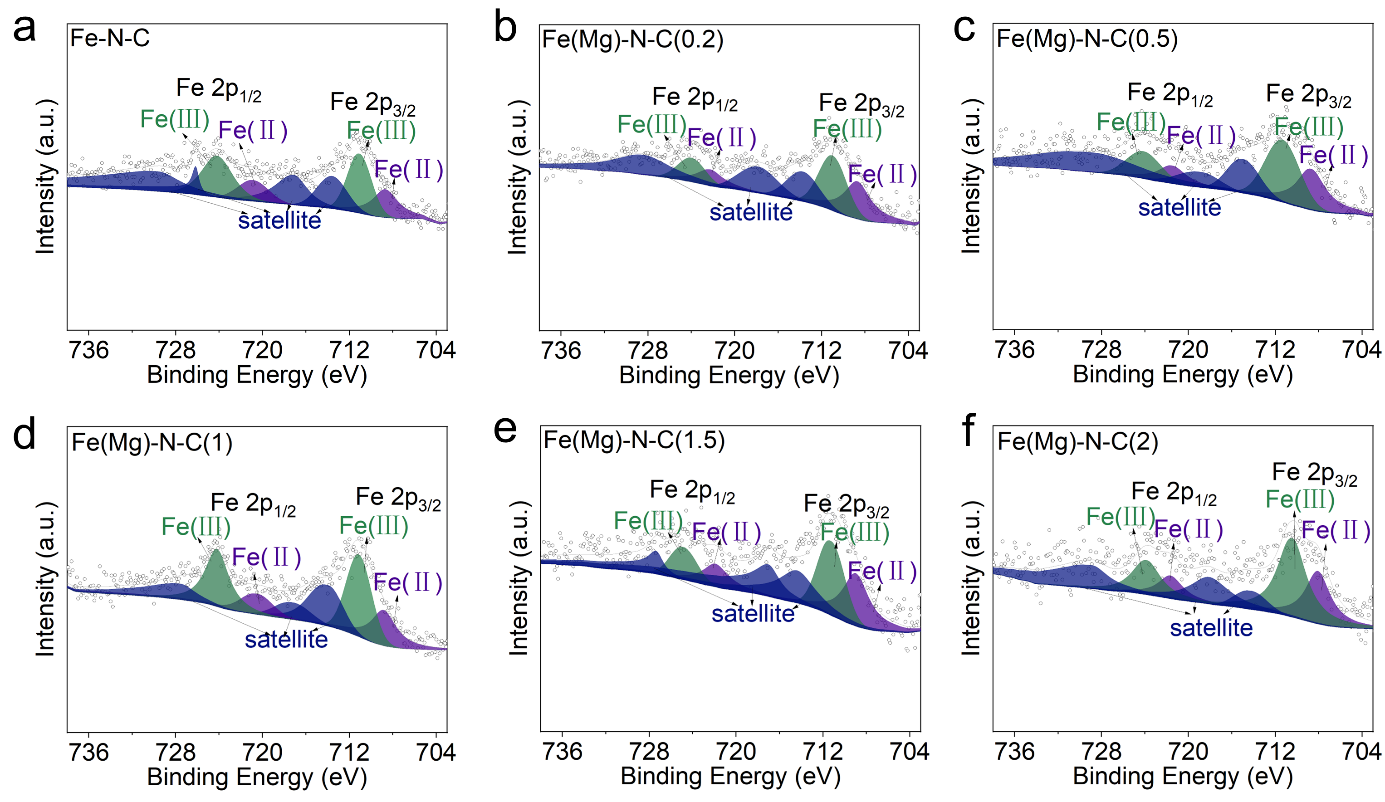


**Figure S15.** (a-f) High-resolution Fe 2p XPS spectra of samples with varying Mg content in the precursor.


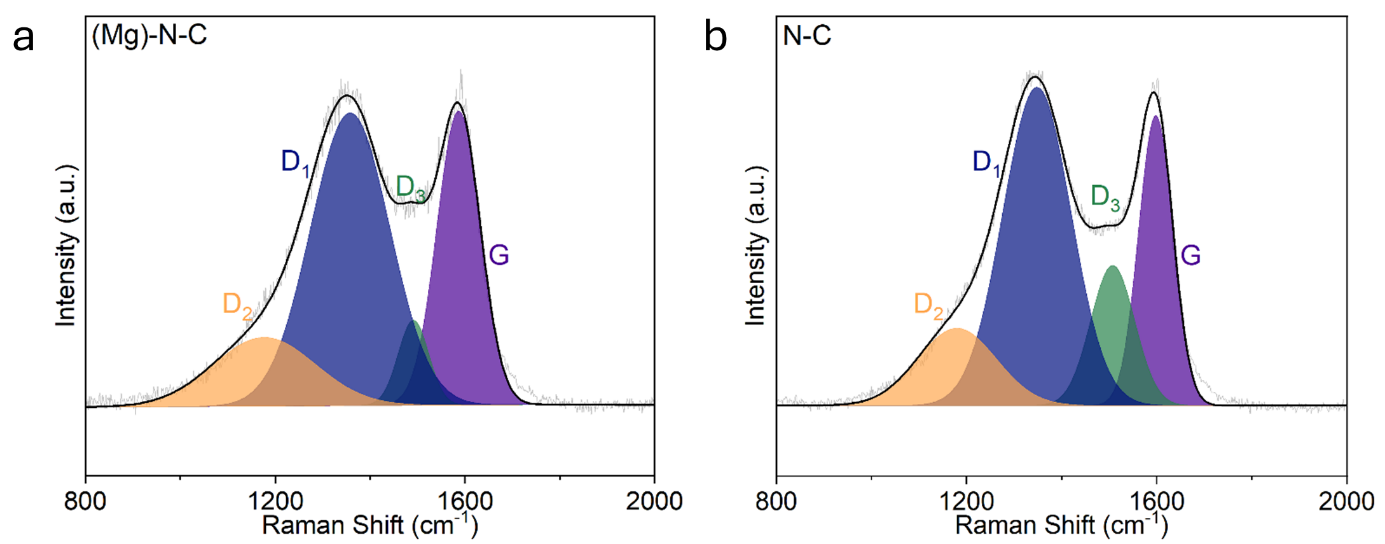


**Figure S16.** (a, b) Raman spectra of (a) (Mg)-N-C and (b) N-C.


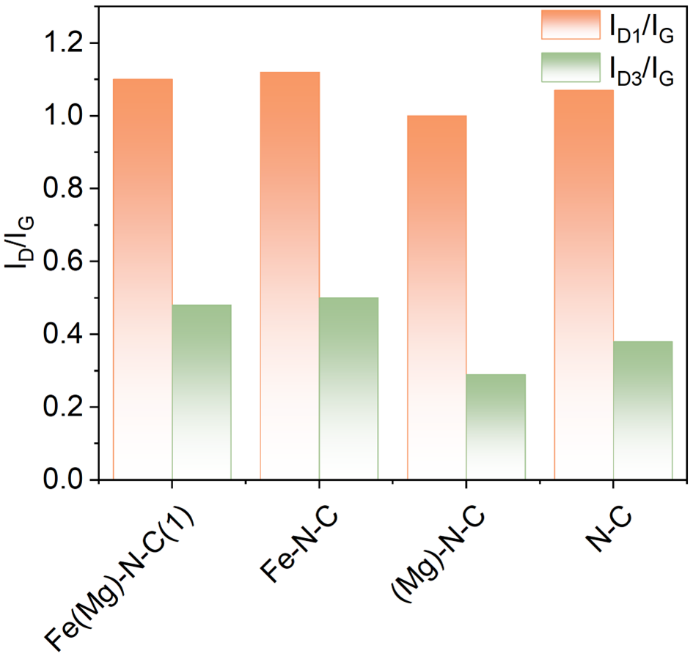


**Figure S17.** I_D1_/I_G_ and I_D3_/I_G_ of Fe(Mg)-N-C(1), Fe-N-C, (Mg)-N-C and N-C catalysts determined by Raman spectra.


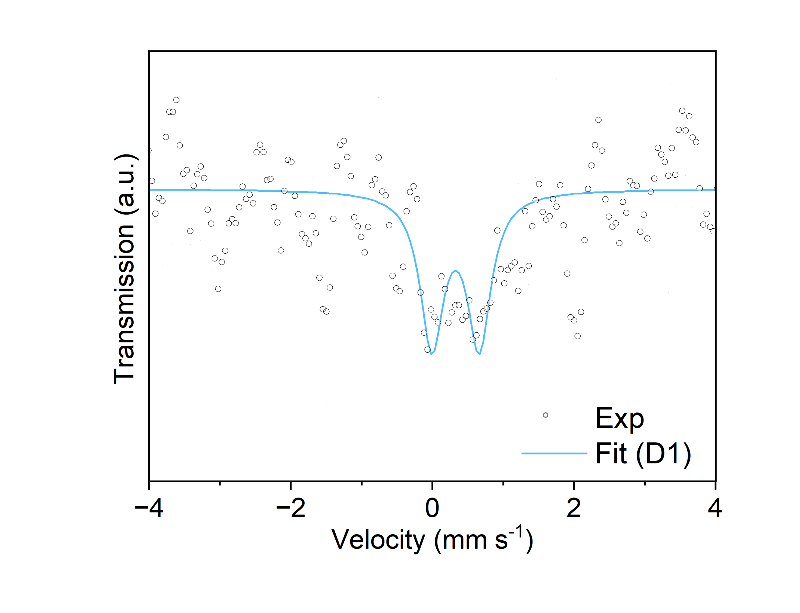


**Figure S18.** ^57^Fe Mössbauer spectra of Fe-N-C at room temperatures.


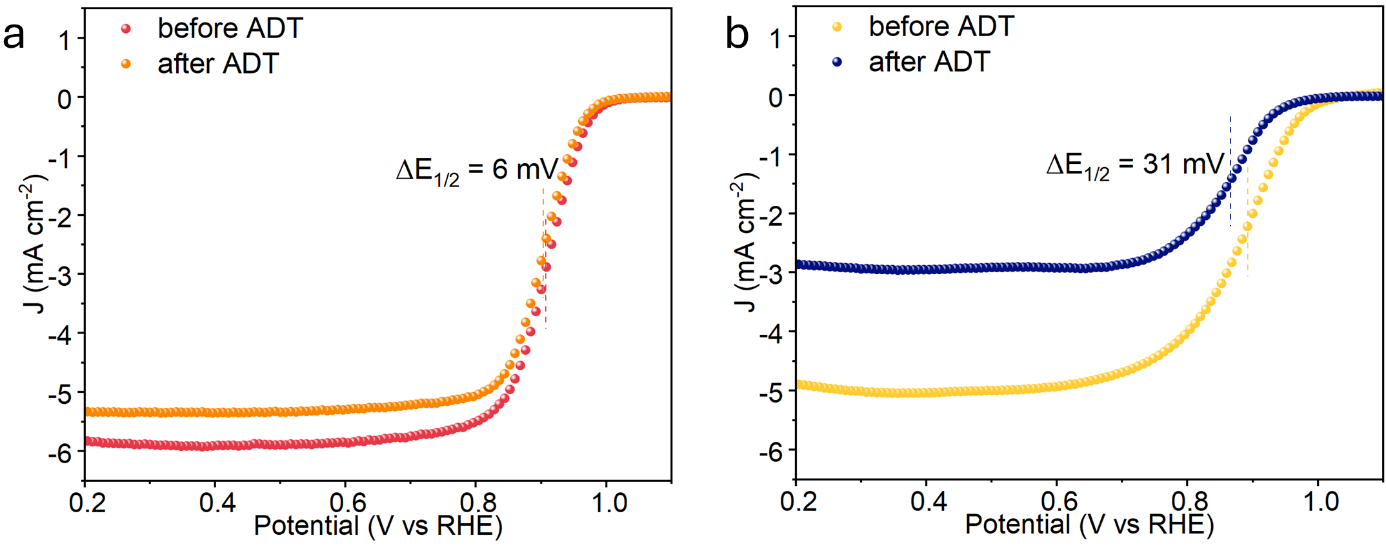


**Figure S19.** (a, b) LSV curves at a sweep rate of 10 mV s^-1^ under O_2_-saturated 0.1 M KOH for (a) Fe(Mg)-N-C(1) and (b) Fe-N-C before and after 5,000 ADT cycles.


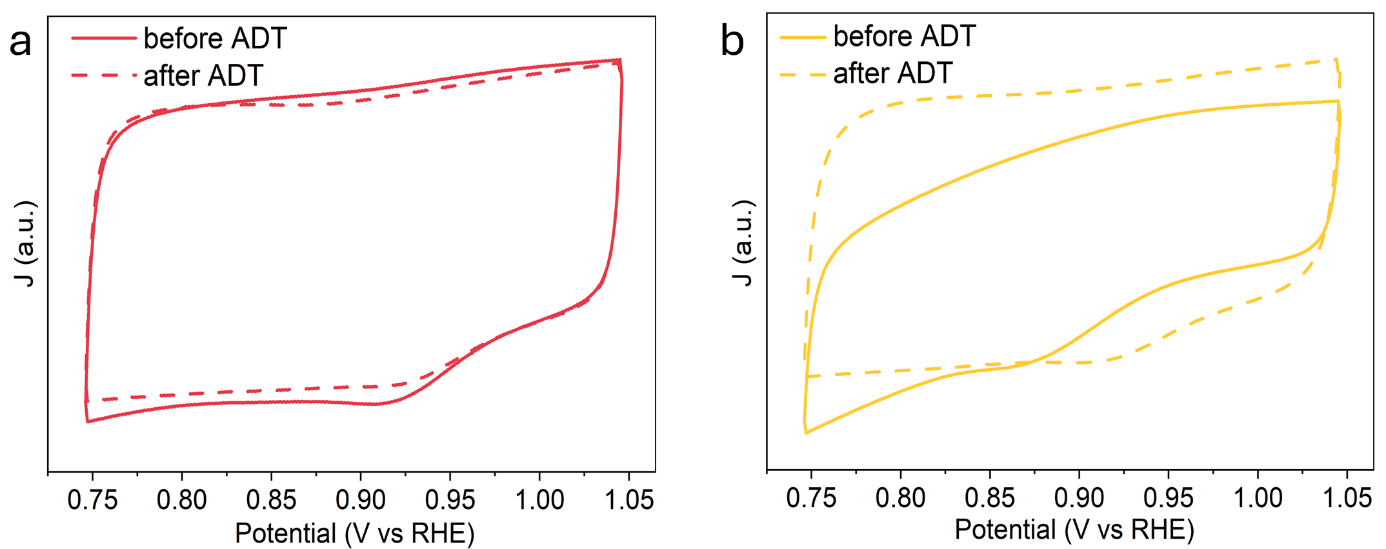


**Figure S20.** (a, b) CV curves between 0.74 and 1.04 V vs. RHE at sweep rate of 100 mV s^-1^ under O_2_-saturated 0.1 M KOH for (a) Fe(Mg)-N-C(1) and (b) Fe-N-C before and after 5,000 ADT cycles.


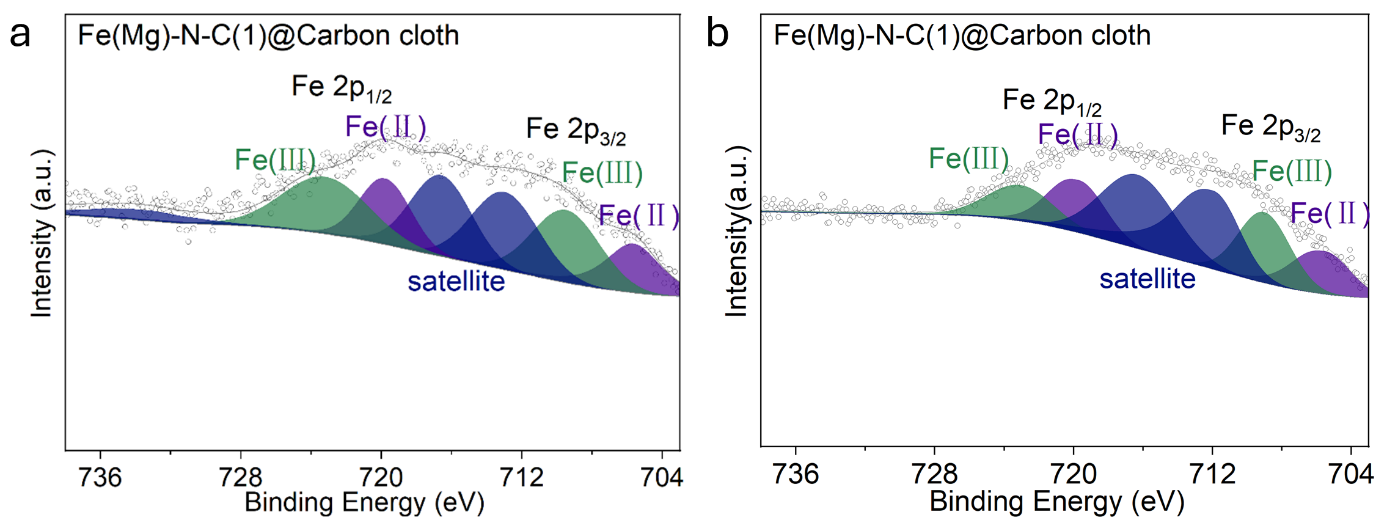


**Figure S21.** (a, b) High-resolution Fe 2p XPS spectra for Fe(Mg)-N-C(1)@carbon cloth catalysts (a) before and (b) after ADT.


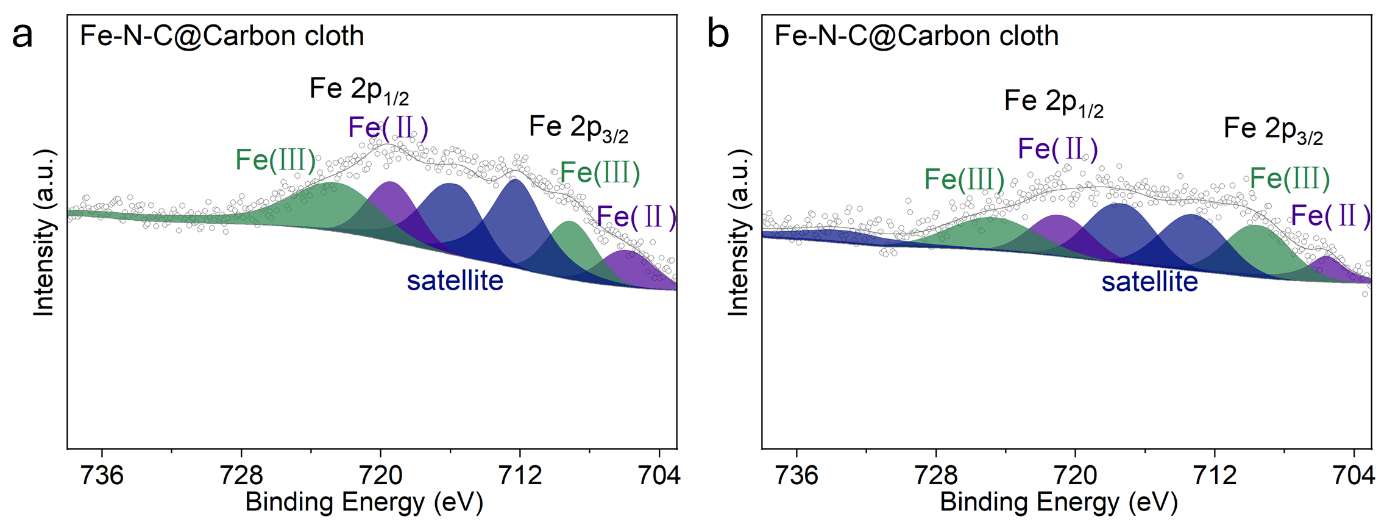


**Figure S22.** (a, b) High-resolution Fe 2p XPS spectra for Fe-N-C@carbon cloth catalysts (a) before and (b) after ADT.


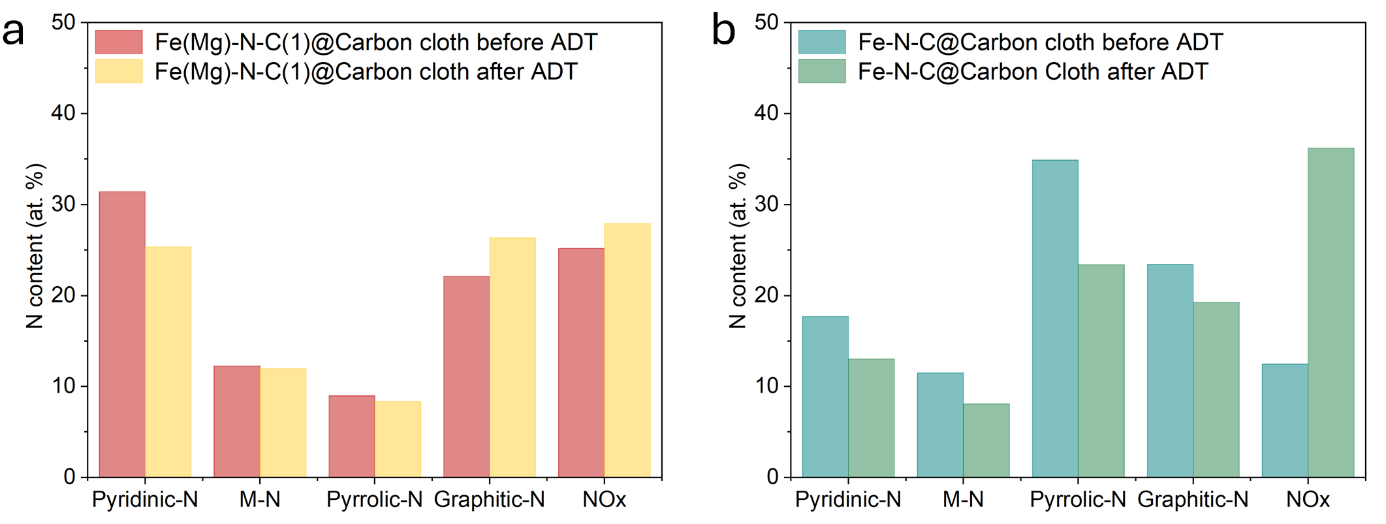


**Figure S23.** (a, b) Surface N compositions (at. %) of (a) Fe(Mg)-N-C(1), (b) Fe-N-C@Carbon cloth before and after ADT.


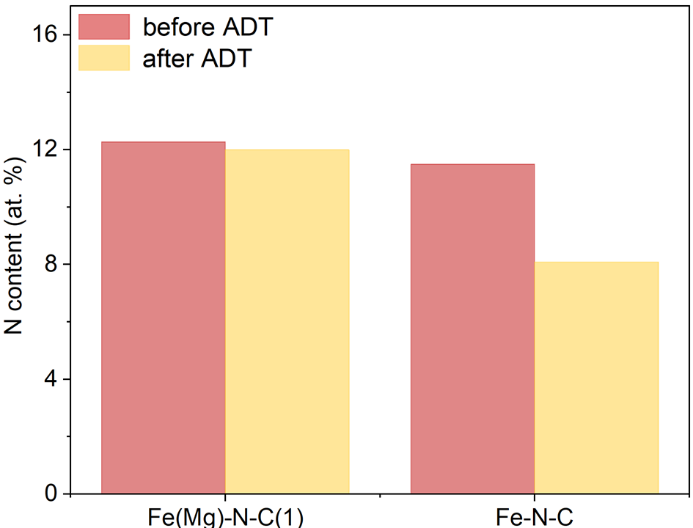


**Figure S24.** M-N content (at. %) of Fe(Mg)-N-C(1) and (Mg)-N-C@Carbon cloth before and after ADT.


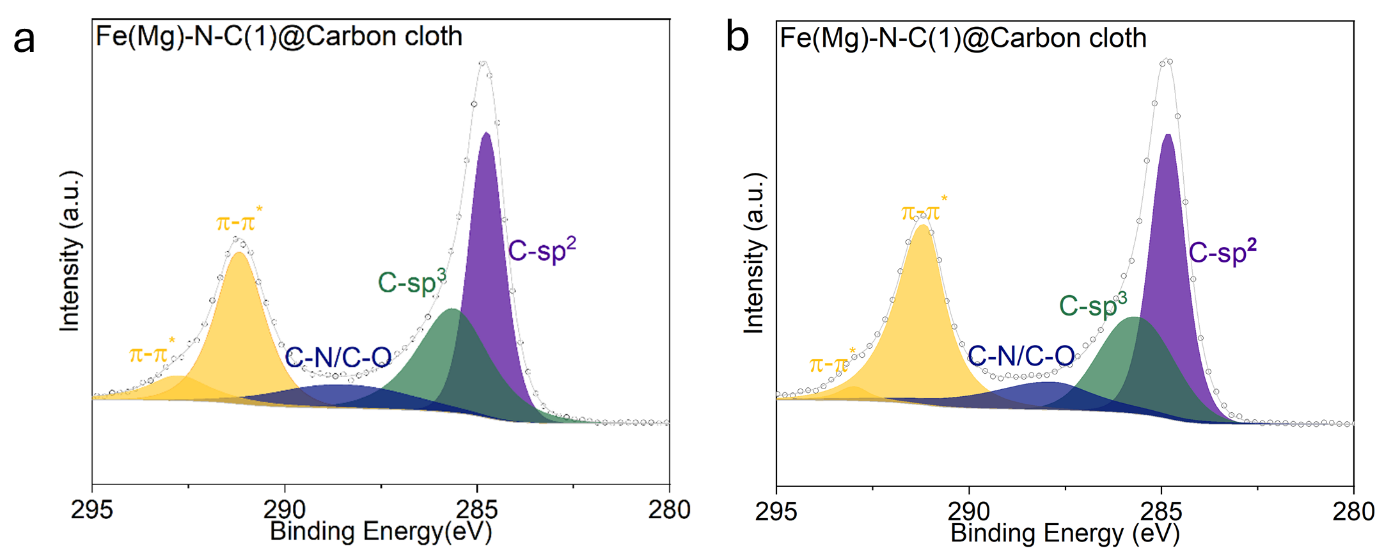


**Figure S25.** (a, b) High-resolution C 1s XPS spectra for Fe(Mg)-N-C(1)@Carbon cloth (a) before and (b) after ADT.


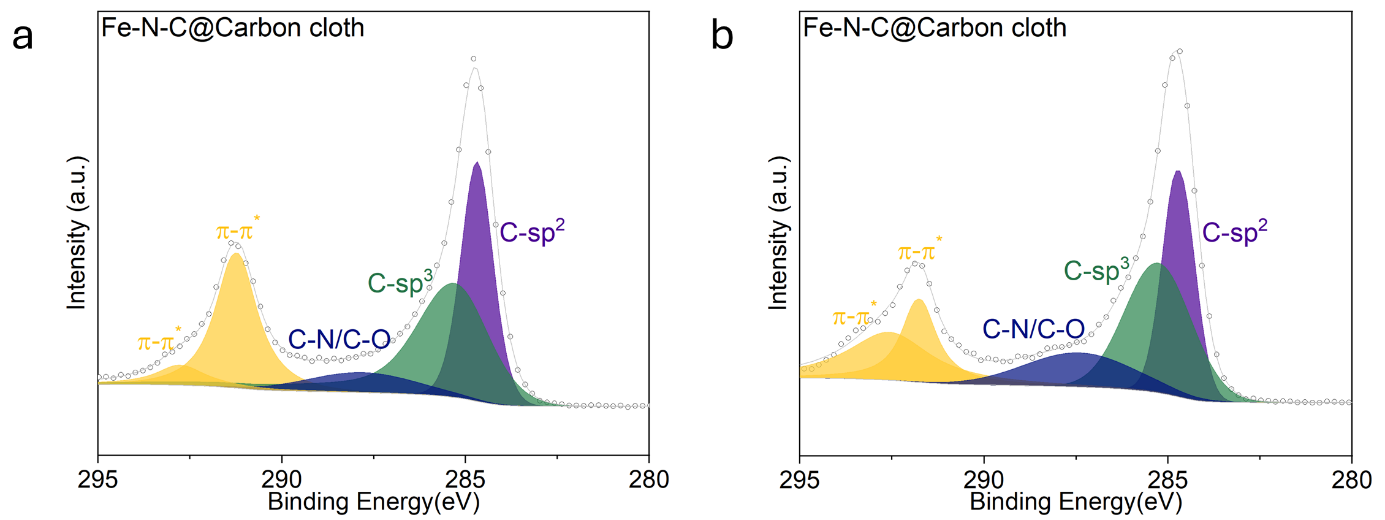


**Figure S26.** (a, b) High-resolution C 1s XPS spectra for Fe-N-C@Carbon cloth (a) before and (b) after ADT.


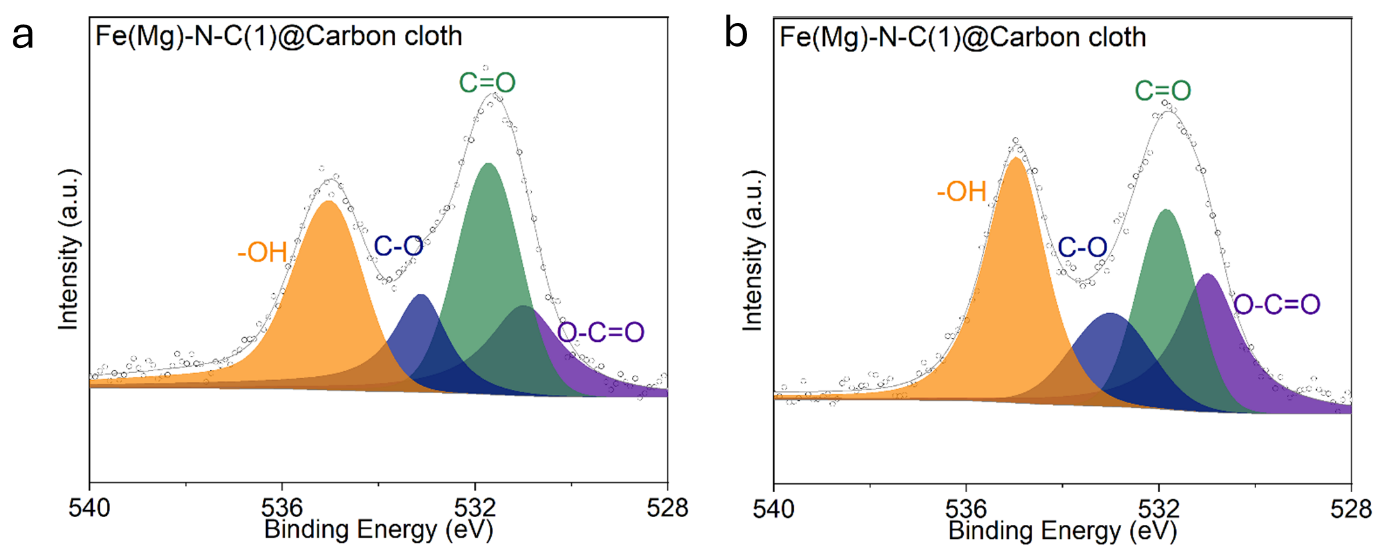


**Figure S27.** (a, b) High-resolution O 1s XPS spectra for Fe(Mg)-N-C(1)@Carbon cloth (a) before and (b) after ADT.


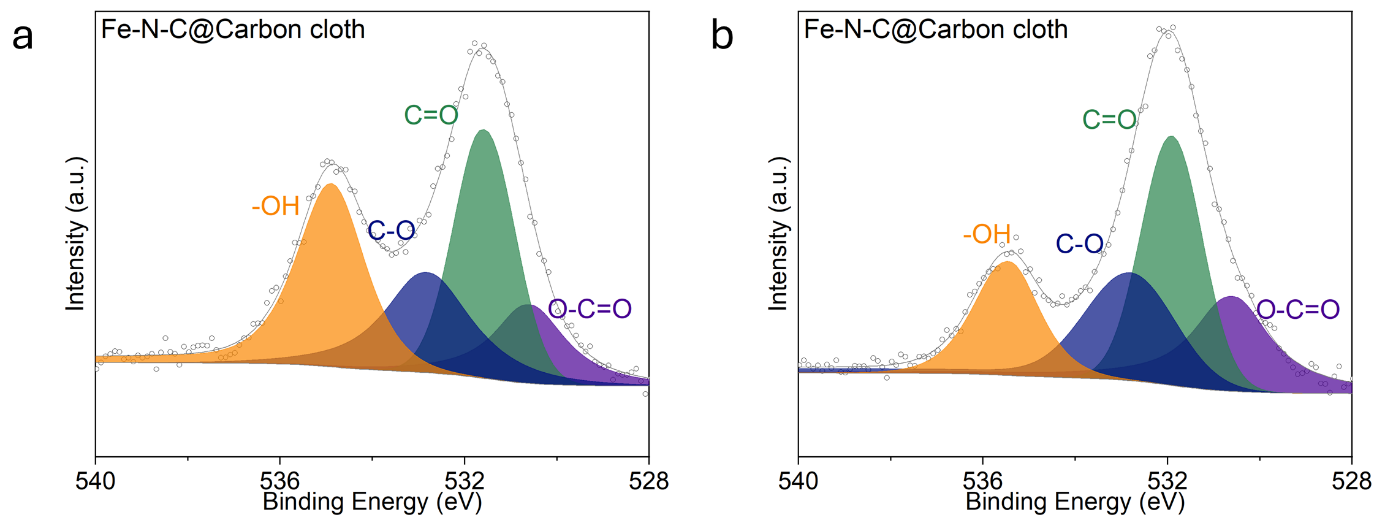


**Figure S28.** (a, b) High-resolution O 1s XPS spectra for Fe-N-C@Carbon cloth (a) before and (b) after ADT.


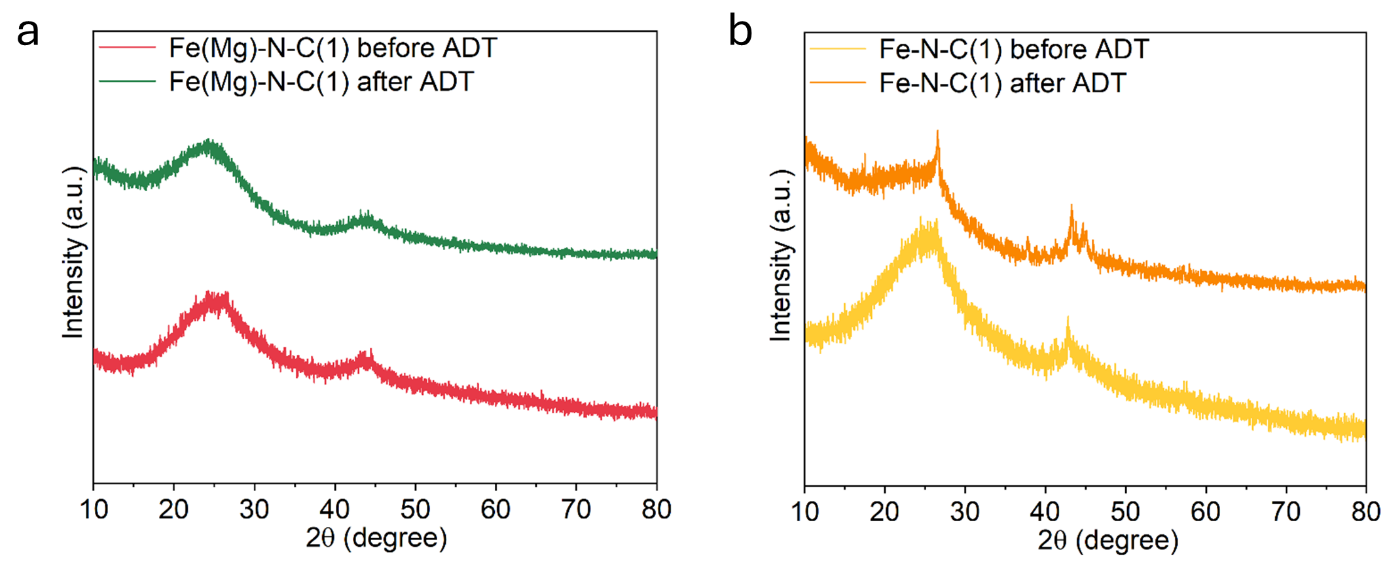


**Figure S29.** XRD patterns of (a) Fe(Mg)-N-C(1), (b) Fe-N-C before and after ADT.


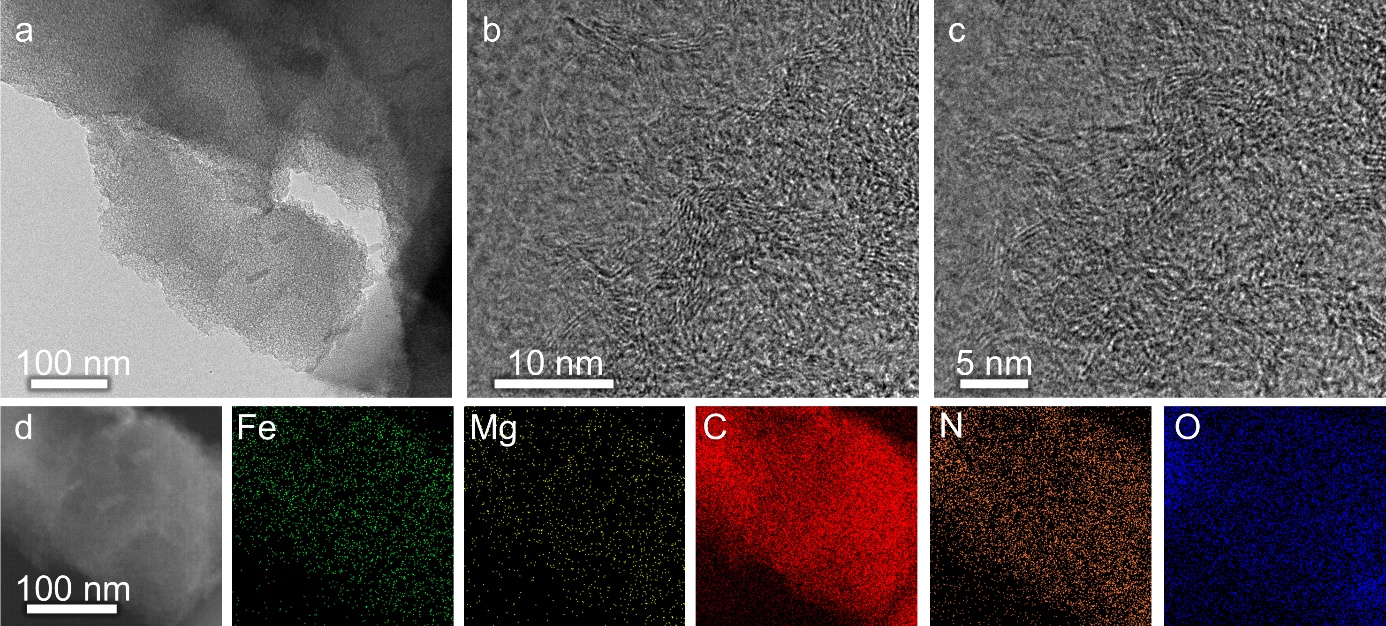


**Figure S30.** (a-c) TEM image and (d) elemental mapping of Fe(Mg)-N-C(1) after ADT.


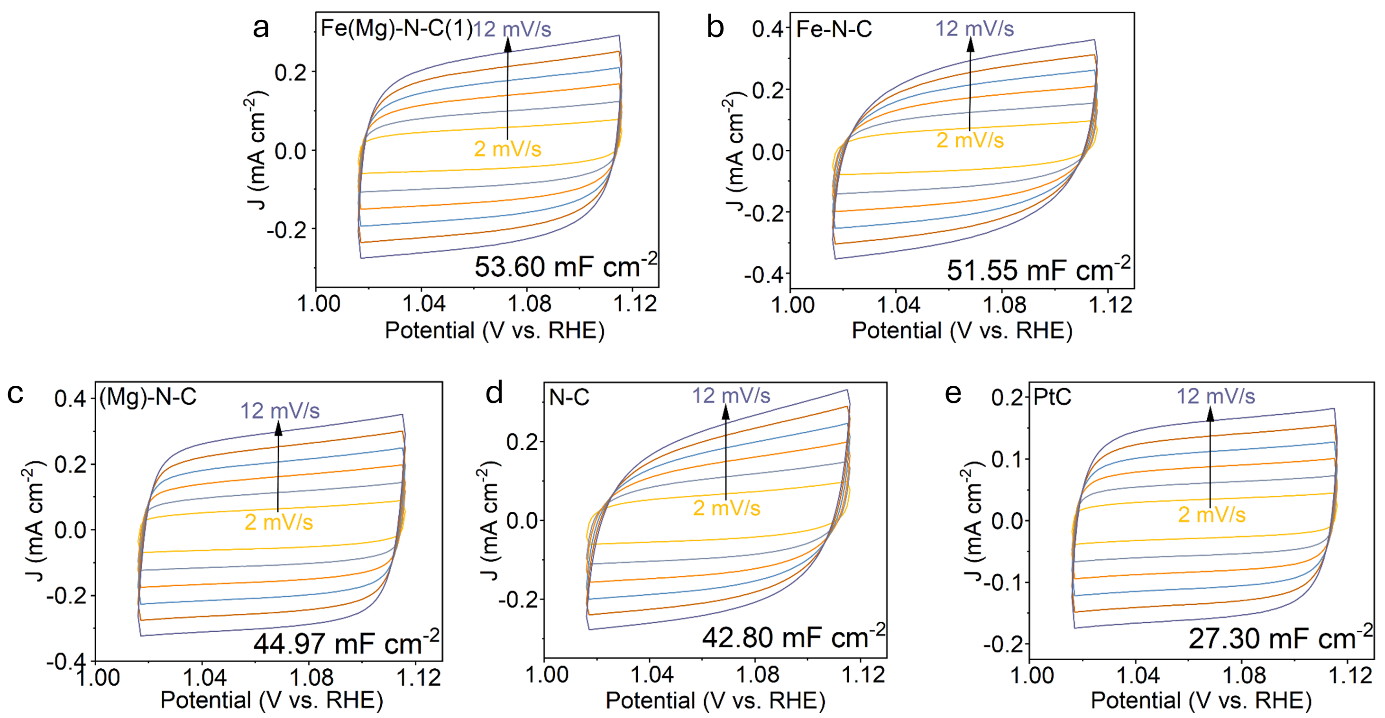


**Figure S31.** (a-e) CV measurement of Fe(Mg)-N-C(1) and reference samples under different scan rates, respectively.


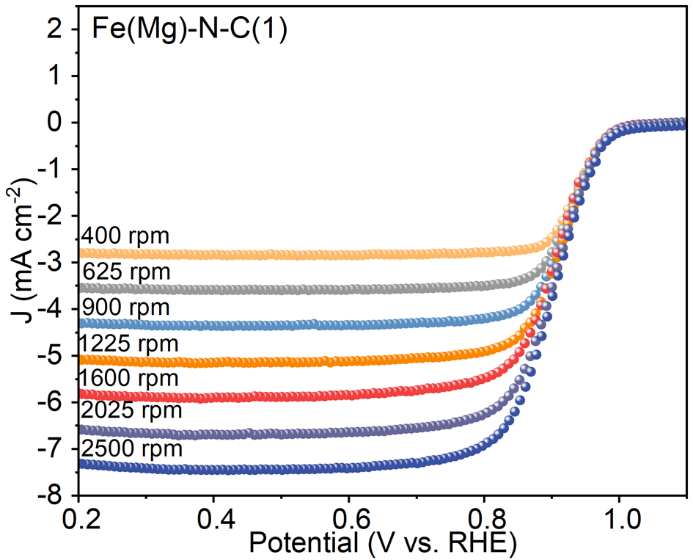


**Figure S32.** ORR polarization curves of Fe(Mg)-N-C(1) at different rotating sweeps.


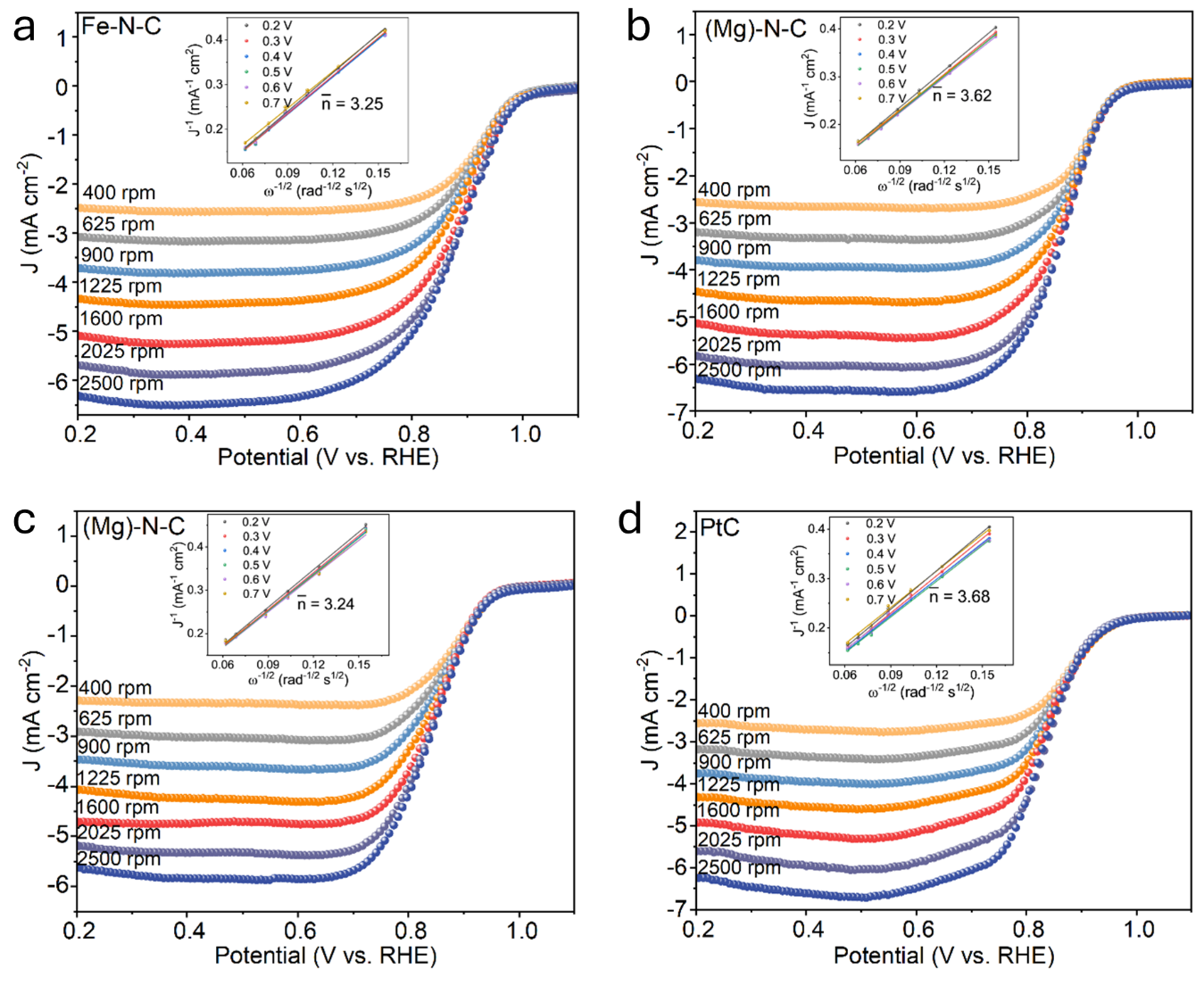


**Figure S33.** (a-d) ORR polarization curves of reference samples at different rotating sweeps in 0.1 M KOH. Insets show the corresponding fitted K-L plots.


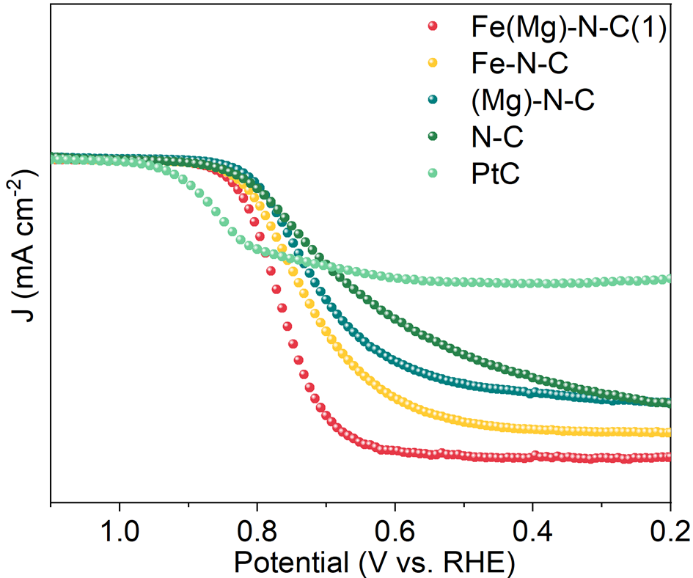


**Figure S34.** LSV curves for Fe(Mg)-N-C(1) and reference samples in O_2_-saturated 0.1 M HClO_4_ at a rotation speed of 1600 rpm.


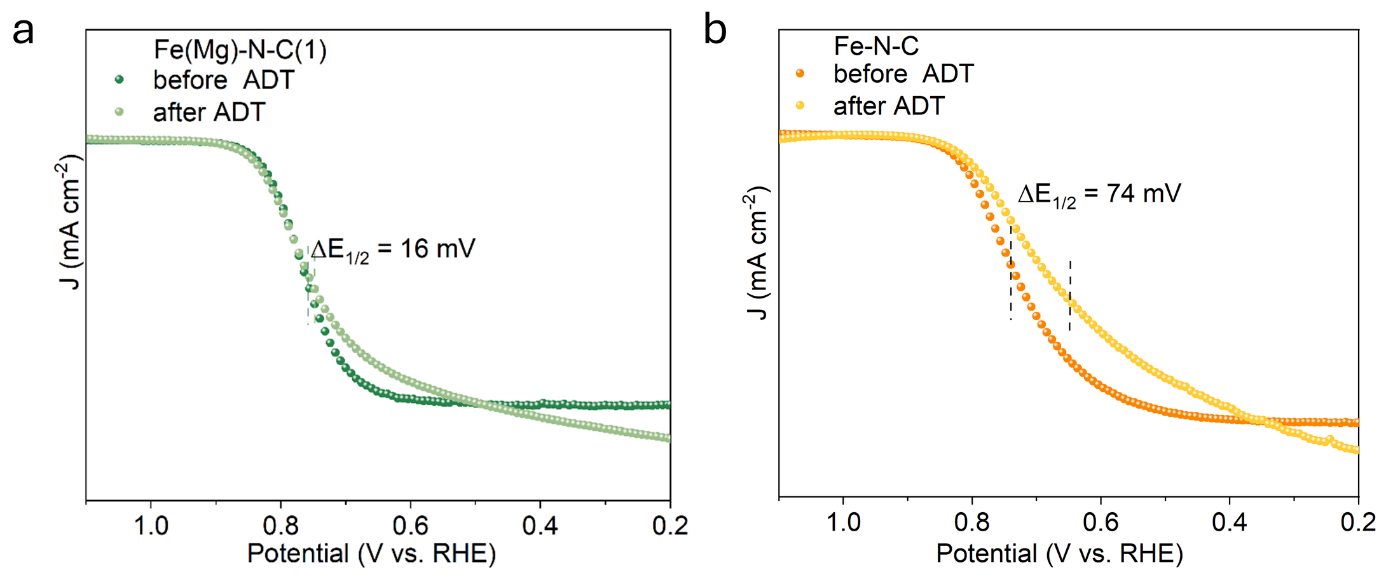


**Figure S35.** (a, b) LSV curves at a sweep rate of 10 mV s^-1^ under O_2_-saturated 0.1 M HClO_4_ for (a) Fe(Mg)-N-C(1) and (b) Fe-N-C before and after 5,000 ADT cycles.


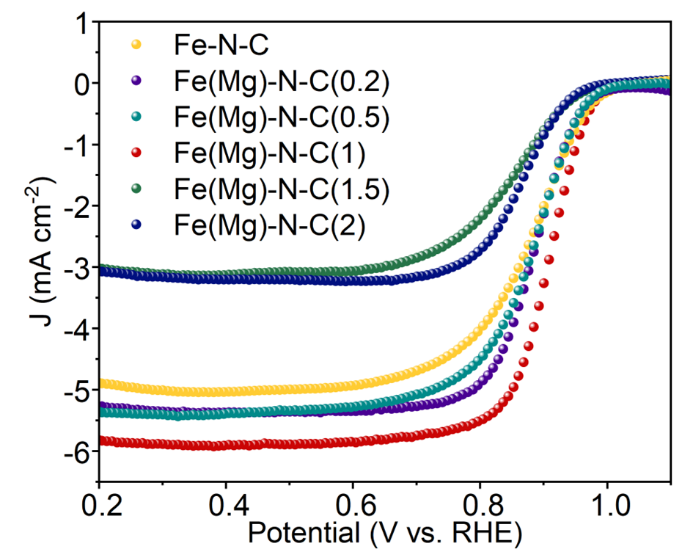


**Figure S36.** LSV curves for samples with varying Mg content in the precursor in O_2_-saturated 0.1 M KOH at a rotation speed of 1600 rpm.


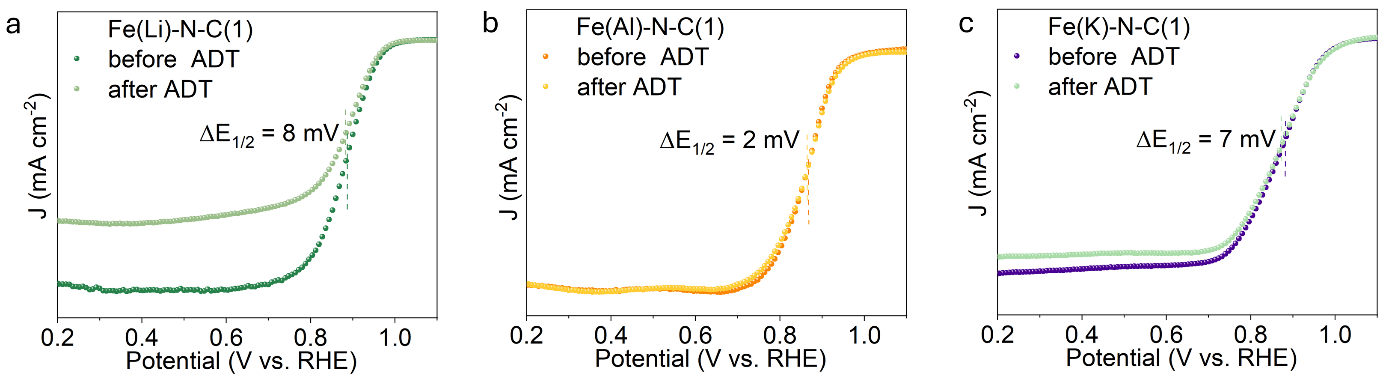


**Figure S37.** (a, b) LSV curves at a sweep rate of 10 mV s^-1^ under O_2_-saturated 0.1 M KOH for (a) Fe(Li)-N-C(1), (b) Fe(Al)-N-C(1) and (c) Fe(K)-N-C(1) before and after 5,000 ADT cycles.


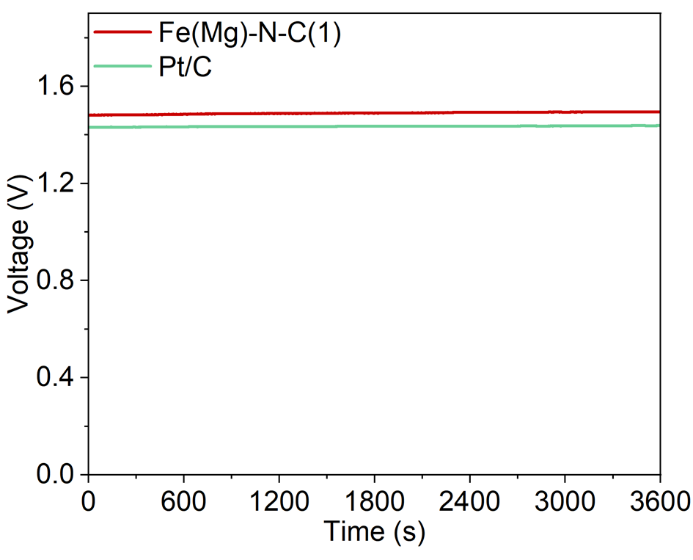


**Figure S38.** OCV of ZABs with Fe(Mg)-N-C(1) and Pt/C cathode.


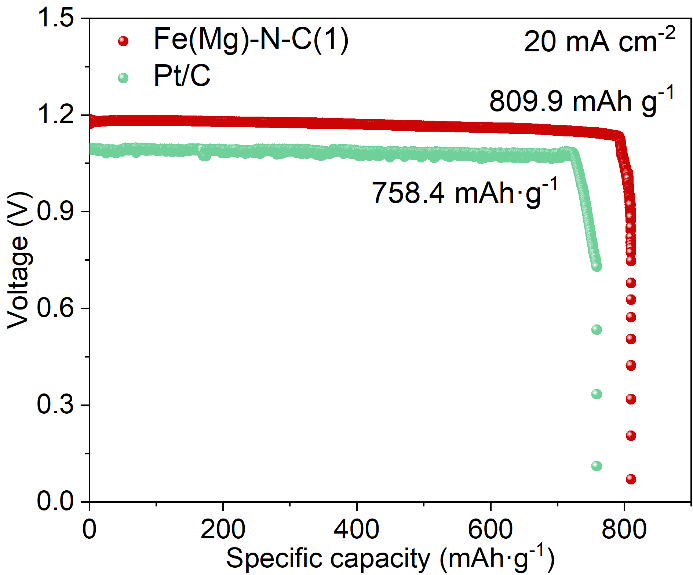


**Figure S39.** Specific capacity diagrams of ZABs with Fe(Mg)-N-C(1) and Pt/C cathode.


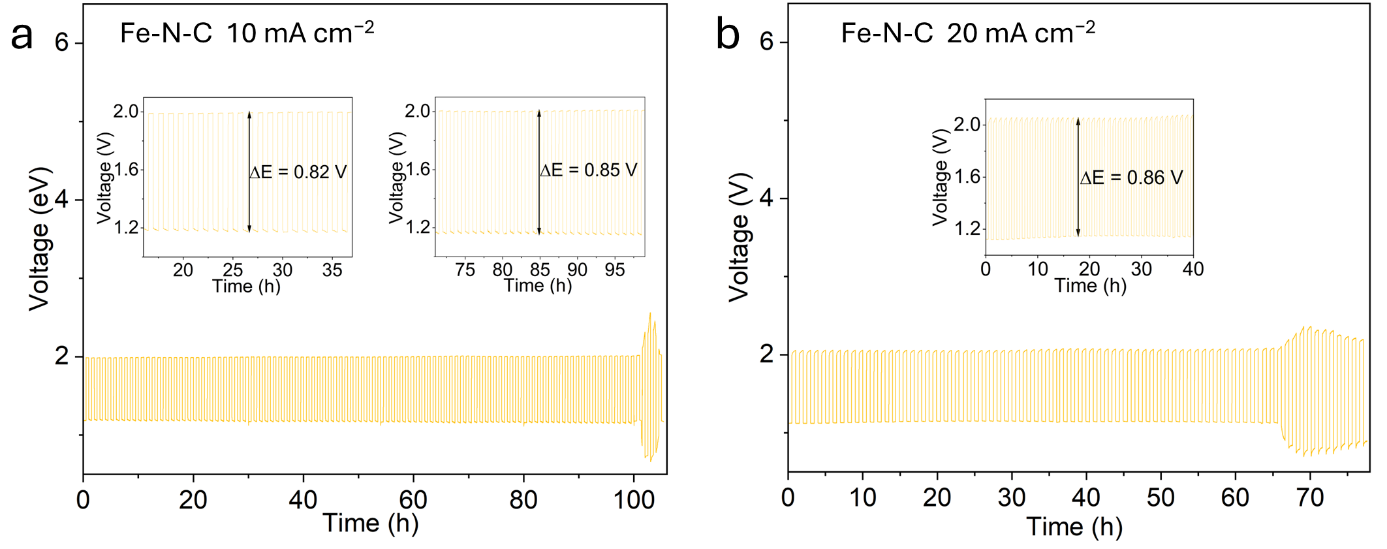


**Figure S40.** (a, b) Cycling performance of ZABs with Fe-N-C cathode at a current density of (a) 10 mA cm^−2^ and (b) 20 mA cm^−2^.

**Supplement Tables**

**Table S1.** Metal loads of Fe(Mg)-N-C(1), Fe-N-C, and (Mg)-N-C catalysts determined by ICP-OES.

| Sample | Element | Content (wt. %) |
| --- | --- | --- |
| Fe(Mg)-N-C(1) | Fe | 3.94 |
|  | Mg | 0.03 |
| Fe-N-C | Fe | 4.02 |
| (Mg)-N-C | Mg | 0.05 |

**Table S2.** Metal loads of the carbonation products of FeMg-IISERP-MOF27 obtained without NH_4_Cl treatment under identical pyrolysis conditions determined by ICP-OES.

| Sample | Element | Content (wt. %) |
| --- | --- | --- |
| Without NH_4_Cl | Fe | 2.85 |
|  | Mg | 1.93 |

**Table S3.** BET surface area of Fe(Mg)-N-C(1), Fe-N-C, (Mg)-N-C and N-C catalysts.

| Sample | BET surface area  (m^2^ g^−1^) | Pore Volume  (cm^3^ g^-1^) | Average pore diameter  (nm) |
| --- | --- | --- | --- |
| Fe(Mg)-N-C(1) | 998.4 | 0.61 | 3.0 |
| Fe-N-C | 1,145.8 | 0.77 | 3.5 |
| (Mg)-N-C | 1,257.8 | 0.62 | 2.3 |
| N-C | 1,465.8 | 0.79 | 2.5 |

**Table S4.** Micro-, and meso-pore ratios of Fe(Mg)-N-C(1), Fe-N-C, (Mg)-N-C and N-C catalysts.

| Sample | Micro-pore ratios  (%) | Meso-pore ratios  (%) |
| --- | --- | --- |
| Fe(Mg)-N-C(1) | 25.4 | 74.6 |
| Fe-N-C | 31.3 | 68.7 |
| (Mg)-N-C | 19.0 | 81.0 |
| N-C | 37.3 | 62.7 |

**Table S5**. Surface compositions (at. %) of Fe(Mg)-N-C(*x*), Fe-N-C, (Mg)-N-C and N-C catalysts by N 1s XPS.

|  | Pyridinic N  (at. %) | M-N  (at. %) | Pyrrolic N  (at. %) | Graphitic N  (at. %) | NO_x_  (at. %) |
| --- | --- | --- | --- | --- | --- |
| Fe-N-C | 13.63 | 19.30 | 15.45 | 22.10 | 29.52 |
| Fe(Mg)-N-C(0.2) | 19.87 | 17.72 | 19.87 | 14.73 | 23.93 |
| Fe(Mg)-N-C(0.4) | 22.36 | 20.06 | 17.59 | 14.93 | 25.06 |
| Fe(Mg)-N-C(1) | 23.68 | 22.55 | 13.56 | 17.04 | 23.17 |
| Fe(Mg)-N-C(1.5) | 30.02 | 15.48 | 10.70 | 14.35 | 29.45 |
| Fe(Mg)-N-C(2) | 30.07 | 13.47 | 10.61 | 20.02 | 25.83 |
| (Mg)-N-C | 30.72 | —— | 17.67 | 33.49 | 18.12 |
| N-C | 20.44 | —— | 39.42 | 9.84 | 30.31 |

**Table S6.** Surface compositions (at. %) of Fe(Mg)-N-C(*x*), Fe-N-C, (Mg)-N-C and N-C catalysts by C 1s XPS.

| Sample | C-sp^2^  (at. %) | C-sp^3^  (at. %) | C-N/C-O  (at. %) | π-π^*^  (at. %) |
| --- | --- | --- | --- | --- |
| Fe-N-C | 39.04 | 43.12 | 9.52 | 8.32 |
| Fe(Mg)-N-C(0.2) | 40.04 | 41.03 | 13.09 | 5.84 |
| Fe(Mg)-N-C(0.4) | 40.32 | 39.91 | 11.96 | 7.82 |
| Fe(Mg)-N-C(1) | 43.04 | 39.57 | 8.79 | 8.60 |
| Fe(Mg)-N-C(1.5) | 50.00 | 33.31 | 8.98 | 7.71 |
| Fe(Mg)-N-C(2) | 50.95 | 30.27 | 9.08 | 9.70 |
| (Mg)-N-C | 39.94 | 39.87 | 13.31 | 6.88 |
| N-C | 37.23 | 43.39 | 11.34 | 8.04 |

**Table S7.** The atomic ratios of the elements in Fe(Mg)-N-C(*x*), Fe-N-C, (Mg)-N-C and N-C catalysts as determined by XPS.

| Sample | C  （at. %） | Fe  （at. %） | Mg  （at. %） | N  （at. %） | O  （at. %） |
| --- | --- | --- | --- | --- | --- |
| Fe-N-C | 85.78 | 0.63 | —— | 5.53 | 8.06 |
| Fe(Mg)-N-C(0.2) | 85.18 | 0.65 | 0.05 | 5.77 | 8.35 |
| Fe(Mg)-N-C(0.5) | 85.74 | 0.63 | 0.17 | 5.91 | 7.55 |
| Fe(Mg)-N-C(1) | 85.12 | 0.64 | 0.3 | 6.23 | 7.71 |
| Fe(Mg)-N-C(1.5) | 83.63 | 0.67 | 0.35 | 7.38 | 7.97 |
| Fe(Mg)-N-C(2) | 83.16 | 0.65 | 0.41 | 7.44 | 8.34 |
| (Mg)-N-C | 85.19 | —— | 0.47 | 6.21 | 8.13 |
| N-C | 86.98 | —— | —— | 4.91 | 8.12 |

**Table S8.** Values of I_D1_/I_G_ and I_D3_/I_G_ of Fe(Mg)-N-C(1), Fe-N-C, (Mg)-N-C and N-C catalysts determined by Raman spectra.

| Sample | I_D1_/I_G_ | I_D3_/I_G_ |
| --- | --- | --- |
| Fe(Mg)-N-C(1) | 1.10 | 0.48 |
| Fe-N-C | 1.12 | 0.50 |
| (Mg)-N-C | 1.00 | 0.29 |
| N-C | 1.07 | 0.38 |

**Table S9.** Surface N compositions (at. %) of Fe(Mg)-N-C(1)@Carbon cloth, Fe-N-C@Carbon cloth before and after ADT.

|  | Pyridinic N  (at. %) | M-N  (at. %) | Pyrrolic N  (at. %) | Graphitic N  (at. %) | NO_x_  (at. %) |
| --- | --- | --- | --- | --- | --- |
| Fe(Mg)-N-C(1)  @Carbon cloth  before ADT | 31.43 | 12.27 | 8.99 | 22.12 | 25.19 |
| Fe(Mg)-N-C(1)  @Carbon cloth  after ADT | 25.36 | 12.00 | 8.34 | 26.37 | 27.93 |
| Fe-N-C  @Carbon cloth  before ADT | 17.70 | 11.49 | 34.91 | 23.43 | 12.47 |
| Fe-N-C  @Carbon cloth  after ADT | 13.03 | 8.09 | 23.39 | 19.28 | 36.21 |

**Table S10.** Tafel slope (mV dec^−1^) of Fe(Mg)-N-C(1), Fe-N-C, (Mg)-N-C and N-C catalysts.

| Sample | Tafel slope  (mV dec^−1^) |
| --- | --- |
| Fe(Mg)-N-C(1) | 47.71 |
| Fe-N-C | 51.64 |
| (Mg)-N-C | 56.44 |
| N-C | 67.04 |
| Pt/C | 66.56 |

**Table S11.** Comparison of ZAB performance between Fe(Mg)-N-C(1) and other reported catalysts as the catalysts for the air cathode.

| Catalysts | E_1/2_  (V vs. RHE) | PPD  (mW cm^−2^) | Current density  (mA cm^−2^) | Stability  (h) | Ref. |
| --- | --- | --- | --- | --- | --- |
| Fe(Mg)-N-C(1) | 0.91 | 271 | 20 | 260 | **This work** |
| FeMn─N─C | 0.92 | 151 | 2 | 210 | **^2^** |
| Fe-Se/NC | 0.925 | 135 | 20 | 200 | **^3^** |
| Fe_SA/AC_@HNC | 0.90 | 171.5 | 5 | 130 | **^4^** |
| Fe_2_N_6_-S | 0.921 | 200.1 | 5 | 550 | **^5^** |
| o-MQFe-10: 20: 5 | 0.861 | 158.2 | 5 | 180 | **^6^** |
| Co_2_/Fe-N@CHC | 0.915 | 232.4 | 10 | 240 | **^7^** |
| FeN_4_-Fe_NCP_@MCF | 0.893 | 208.1 | 5 | 350 | **^8^** |
| FeMn-DSAC | 0.922 | 184 | 2 | 80 | **^9^** |
| Fe SAs HS | 0.86 | 170 | 20 | 65 | **^10^** |
| PCF-FeTz-900 | 0.85 | 180 | 10 | 90 | **^11^** |
| Fe-N_2_-Fe DAC | 0.91 | 169.8 | 10 | 400 | **^12^** |
| Fe-SA/N-HCS | 0.91 | 191 | 5 | 400 | **^13^** |
| Fe-N/S-C | 0.882 | 203 | 20 | 70 | **^14^** |

**References**

(1) Liu, Y.; Yuan, S.; Sun, C.; Wang, C.; Liu, X.; Lv, Z.; Liu, R.; Meng, Y.; Yang, W.; Feng, X.; Wang, B. Optimizing Fe-3d Electron Delocalization by Asymmetric Fe–Cu Diatomic Configurations for Efficient Anion Exchange Membrane Fuel Cells. *Adv. Energy Mater.* **2023**, *13*, 2302719.

(2) Hu, C.; Xing, G.; Han, W.; Hao, Y.; Zhang, C.; Zhang, Y.; Kuo, C. H.; Chen, H. Y.; Hu, F.; Li, L.; Peng, S. Inhibiting Demetalation of Fe−N−C via Mn Sites for Efficient Oxygen Reduction Reaction in Zinc-Air Batteries. *Adv. Mater.* **2024**, *36*, 2405763.

(3) Wang, Y.; Wu, J.; Tang, S.; Yang, J.; Ye, C.; Chen, J.; Lei, Y.; Wang, D. Synergistic Fe−Se Atom Pairs as Bifunctional Oxygen Electrocatalysts Boost Low-Temperature Rechargeable Zn-Air Battery. *Angew. Chem. Int. Ed.* **2023**, *62*, e202219191.

(4) Zhang, H.; Chen, H. C.; Feizpoor, S.; Li, L.; Zhang, X.; Xu, X.; Zhuang, Z.; Li, Z.; Hu, W.; Snyders, R.; Wang, D.; Wang, C. Tailoring Oxygen Reduction Reaction Kinetics of Fe−N−C Catalyst via Spin Manipulation for Efficient Zinc–Air Batteries. *Adv. Mater.* **2024**, *36*, 2400523.

(5) Liu, M.; Wang, X.; Cao, S.; Lu, X.; Li, W.; Li, N.; Bu, X. H. Ferredoxin-Inspired Design of S-Synergized Fe–Fe Dual-Metal Center Catalysts for Enhanced Electrocatalytic Oxygen Reduction Reaction. *Adv. Mater.* **2024**, *36*, 2309231.

(6) Liu, Y.; Liu, X.; Lv, Z.; Liu, R.; Li, L.; Wang, J.; Yang, W.; Jiang, X.; Feng, X.; Wang, B. Tuning the Spin State of the Iron Center by Bridge-Bonded Fe-O-Ti Ligands for Enhanced Oxygen Reduction. *Angew. Chem. Int. Ed.* **2022**, *61*, e202117617.

(7) Wang, Z.; Jin, X.; Zhu, C.; Liu, Y.; Tan, H.; Ku, R.; Zhang, Y.; Zhou, L.; Liu, Z.; Hwang, S. J.; Fan, H. J. Atomically Dispersed Co_2_–N_6_ and Fe–N_4_ Costructures Boost Oxygen Reduction Reaction in Both Alkaline and Acidic Media. *Adv. Mater.* **2021**, *33*, 2104718.

(8) Wang, Z.; Lu, Z.; Ye, Q.; Yang, Z.; Xu, R.; Kong, K.; Zhang, Y.; Yan, T.; Liu, Y.; Pan, Z.; Huang, Y.; Lu, X. Construction of Fe Nanoclusters/Nanoparticles to Engineer FeN_4_ Sites on Multichannel Porous Carbon Fibers for Boosting Oxygen Reduction Reaction. *Adv. Funct. Mater.* **2024**, *34*, 2315150.

(9) Cui, T.; Wang, Y. P.; Ye, T.; Wu, J.; Chen, Z.; Li, J.; Lei, Y.; Wang, D.; Li, Y. Engineering Dual Single-Atom Sites on 2D Ultrathin N-doped Carbon Nanosheets Attaining Ultra-Low-Temperature Zinc-Air Battery. *Angew. Chem. Int. Ed.* **2022**, *61*, e202115219.

(10) Wang, Y.; Meng, P.; Yang, Z.; Jiang, M.; Yang, J.; Li, H.; Zhang, J.; Sun, B.; Fu, C. Regulation of Atomic Fe-Spin State by Crystal Field and Magnetic Field for Enhanced Oxygen Electrocatalysis in Rechargeable Zinc-Air Batteries. *Angew. Chem. Int. Ed.* **2023**, *62*, e202304229.

(11) Qin, Y.; Ou, Z.; Guo, C.; Liu, Y.; Jin, R.; Xu, C.; Chen, H.; Si, Y.; Li, H. Phosphor-doping modulates the d-band center of Fe atoms in Fe-N_4_ catalytic sites to boost the activity of oxygen reduction. *Appl. Catal. B Environ. Energy* **2024**, *360*, 124553.

(12) Zhao, S.; Liu, M.; Qu, Z.; Yan, Y.; Zhang, Z.; Yang, J.; He, S.; Xu, Z.; Zhu, Y.; Luo, L.; Hui, K. N.; Liu, M.; Zeng, J. Cascade Synthesis of Fe-N_2_-Fe Dual-Atom Catalysts for Superior Oxygen Catalysis. *Angew. Chem. Int. Ed.* **2024**, *63*, e202408914.

(13) Zong, L.; Fan, K.; Cui, L.; Lu, F.; Liu, P.; Li, B.; Feng, S.; Wang, L. Constructing Fe-N_4_ Sites through Anion Exchange-mediated Transformation of Fe Coordination Environments in Hierarchical Carbon Support for Efficient Oxygen Reduction. *Angew. Chem. Int. Ed.* **2023**, *62*, e202309784.

(14) Li, L.; Huang, S.; Cao, R.; Yuan, K.; Lu, C.; Huang, B.; Tang, X.; Hu, T.; Zhuang, X.; Chen, Y. Optimizing Microenvironment of Asymmetric N,S-Coordinated Single-Atom Fe via Axial Fifth Coordination toward Efficient Oxygen Electroreduction. *Small* **2021**, *18*, 2105387.
